# Machine Learning to Predict Mortality and Critical Events in a Cohort of Patients With COVID-19 in New York City: Model Development and Validation

**DOI:** 10.2196/24018

**Published:** 2020-11-06

**Authors:** Akhil Vaid, Sulaiman Somani, Adam J Russak, Jessica K De Freitas, Fayzan F Chaudhry, Ishan Paranjpe, Kipp W Johnson, Samuel J Lee, Riccardo Miotto, Felix Richter, Shan Zhao, Noam D Beckmann, Nidhi Naik, Arash Kia, Prem Timsina, Anuradha Lala, Manish Paranjpe, Eddye Golden, Matteo Danieletto, Manbir Singh, Dara Meyer, Paul F O'Reilly, Laura Huckins, Patricia Kovatch, Joseph Finkelstein, Robert M. Freeman, Edgar Argulian, Andrew Kasarskis, Bethany Percha, Judith A Aberg, Emilia Bagiella, Carol R Horowitz, Barbara Murphy, Eric J Nestler, Eric E Schadt, Judy H Cho, Carlos Cordon-Cardo, Valentin Fuster, Dennis S Charney, David L Reich, Erwin P Bottinger, Matthew A Levin, Jagat Narula, Zahi A Fayad, Allan C Just, Alexander W Charney, Girish N Nadkarni, Benjamin S Glicksberg

**Affiliations:** 1 The Hasso Plattner Institute for Digital Health at Mount Sinai Icahn School of Medicine at Mount Sinai New York, NY United States; 2 Department of Medicine Icahn School of Medicine at Mount Sinai New York, NY United States; 3 Department of Genetics and Genomic Sciences Icahn School of Medicine at Mount Sinai New York, NY United States; 4 Department of Anesthesiology, Perioperative and Pain Medicine Icahn School of Medicine at Mount Sinai New York, NY United States; 5 Department of Population Health Science and Policy Icahn School of Medicine at Mount Sinai New York, NY United States; 6 Institute for Healthcare Delivery Science Icahn School of Medicine at Mount Sinai New York, NY United States; 7 The Zena and Michael A. Wiener Cardiovascular Institute Icahn School of Medicine at Mount Sinai New York, NY United States; 8 Harvard Medical School Boston, MA United States; 9 The Pamela Sklar Division of Psychiatric Genomics Icahn School of Medicine at Mount Sinai New York, NY United States; 10 The Department of Psychiatry Icahn School of Medicine at Mount Sinai New York, NY United States; 11 Mount Sinai Data Warehouse Icahn School of Medicine at Mount Sinai New York, NY United States; 12 Mount Sinai Heart Icahn School of Medicine at Mount Sinai New York, NY United States; 13 Department of Cardiology Icahn School of Medicine at Mount Sinai New York, NY United States; 14 Icahn Institute for Data Science and Genomic Technology Icahn School of Medicine at Mount Sinai New York, NY United States; 15 Mount Sinai Data Office Icahn School of Medicine at Mount Sinai New York, NY United States; 16 Division of Infectious Diseases Icahn School of Medicine at Mount Sinai New York, NY United States; 17 Nash Family Department of Neuroscience Icahn School of Medicine at Mount Sinai New York, NY United States; 18 Friedman Brain Institute Icahn School of Medicine at Mount Sinai New York, NY United States; 19 The Charles Bronfman Institute for Personalized Medicine Icahn School of Medicine at Mount Sinai New York, NY United States; 20 Department of Pathology Icahn School of Medicine at Mount Sinai New York, NY United States; 21 Office of the Dean Icahn School of Medicine at Mount Sinai New York, NY United States; 22 Digital Health Center Hasso Plattner Institute University of Potsdam Potsdam Germany; 23 BioMedical Engineering and Imaging Institute Icahn School of Medicine at Mount Sinai New York, NY United States; 24 Department of Radiology Icahn School of Medicine at Mount Sinai New York, NY United States; 25 Department of Environmental Medicine and Public Health Icahn School of Medicine at Mount Sinai New York, NY United States

**Keywords:** machine learning, COVID-19, electronic health record, TRIPOD, clinical informatics, prediction, mortality, EHR, cohort, hospital, performance

## Abstract

**Background:**

COVID-19 has infected millions of people worldwide and is responsible for several hundred thousand fatalities. The COVID-19 pandemic has necessitated thoughtful resource allocation and early identification of high-risk patients. However, effective methods to meet these needs are lacking.

**Objective:**

The aims of this study were to analyze the electronic health records (EHRs) of patients who tested positive for COVID-19 and were admitted to hospitals in the Mount Sinai Health System in New York City; to develop machine learning models for making predictions about the hospital course of the patients over clinically meaningful time horizons based on patient characteristics at admission; and to assess the performance of these models at multiple hospitals and time points.

**Methods:**

We used Extreme Gradient Boosting (XGBoost) and baseline comparator models to predict in-hospital mortality and critical events at time windows of 3, 5, 7, and 10 days from admission. Our study population included harmonized EHR data from five hospitals in New York City for 4098 COVID-19–positive patients admitted from March 15 to May 22, 2020. The models were first trained on patients from a single hospital (n=1514) before or on May 1, externally validated on patients from four other hospitals (n=2201) before or on May 1, and prospectively validated on all patients after May 1 (n=383). Finally, we established model interpretability to identify and rank variables that drive model predictions.

**Results:**

Upon cross-validation, the XGBoost classifier outperformed baseline models, with an area under the receiver operating characteristic curve (AUC-ROC) for mortality of 0.89 at 3 days, 0.85 at 5 and 7 days, and 0.84 at 10 days. XGBoost also performed well for critical event prediction, with an AUC-ROC of 0.80 at 3 days, 0.79 at 5 days, 0.80 at 7 days, and 0.81 at 10 days. In external validation, XGBoost achieved an AUC-ROC of 0.88 at 3 days, 0.86 at 5 days, 0.86 at 7 days, and 0.84 at 10 days for mortality prediction. Similarly, the unimputed XGBoost model achieved an AUC-ROC of 0.78 at 3 days, 0.79 at 5 days, 0.80 at 7 days, and 0.81 at 10 days. Trends in performance on prospective validation sets were similar. At 7 days, acute kidney injury on admission, elevated LDH, tachypnea, and hyperglycemia were the strongest drivers of critical event prediction, while higher age, anion gap, and C-reactive protein were the strongest drivers of mortality prediction.

**Conclusions:**

We externally and prospectively trained and validated machine learning models for mortality and critical events for patients with COVID-19 at different time horizons. These models identified at-risk patients and uncovered underlying relationships that predicted outcomes.

## Introduction

Despite substantial, organized efforts to prevent disease spread, over 23 million people have tested positive for SARS-CoV-2 worldwide, and the World Health Organization has reported more than 800,000 deaths from the virus to date [1-4]. As a result of this pandemic, hospitals are being filled beyond capacity and face extreme challenges with regard to personnel staffing, personal protective equipment availability, and intensive care unit (ICU) bed allocation. Additionally, patients with COVID-19 demonstrate varying symptomatology, making safe and successful patient triaging difficult. While some infected patients are asymptomatic, others suffer from severe acute respiratory distress syndrome, experience multiorgan failure, or die [5-7]. Identification of key patient characteristics that govern the course of disease across large patient cohorts is important, particularly given its potential to aid physicians and hospitals in predicting disease trajectory, allocating essential resources effectively, and improving patient outcomes. Prognostication with machine learning is poised to accomplish this [8]; however, efforts have been limited by small sample sizes, lack of generalization to diverse populations, disparities in feature missingness, and potential for bias [9]. Many predictive models have met with success; however, these models only consider demographics, clinical symptoms, or laboratory values rather than considering all these factors conjointly [10-17]. More recent studies have accounted for fundamental aspects of machine learning but are limited in scope [13,18-22]. These studies lack either temporal benchmarks, interhospital or prospective validation, systematic evaluation of multiple models, consideration of covariate correlations, or assessment of the impact of the imputed data. With these needs in mind, we report the development of a boosted decision tree–based machine learning model trained on electronic health records from patients confirmed to have COVID-19 at a single center in the Mount Sinai Health System (MSHS) in New York City to predict critical events and mortality. To assess both interhospital and temporal generalizability, we first externally validated this algorithm to four other hospital centers. We then prospectively validated it on a new set of patients from all five hospitals. Finally, we performed a saliency analysis using SHAP (SHapley Additive exPlanation) values to identify the most important features used by this model for outcome prediction.

## Methods

### Clinical Data Sources

Patient data were obtained from five hospitals within the MSHS in New York City: the Mount Sinai Hospital (MSH) located in East Harlem, Manhattan; Mount Sinai Morningside (MSM) located in Morningside Heights, Manhattan; Mount Sinai West (MSW) located in Midtown West, Manhattan; Mount Sinai Brooklyn (MSB) located in Midwood, Brooklyn; and Mount Sinai Queens (MSQ) located in Astoria, Queens. The data set was obtained from different sources using the Epic EHR software (Epic Systems) and aggregated by the Mount Sinai COVID Informatics Center.

### Study Population

**Figure 1:**
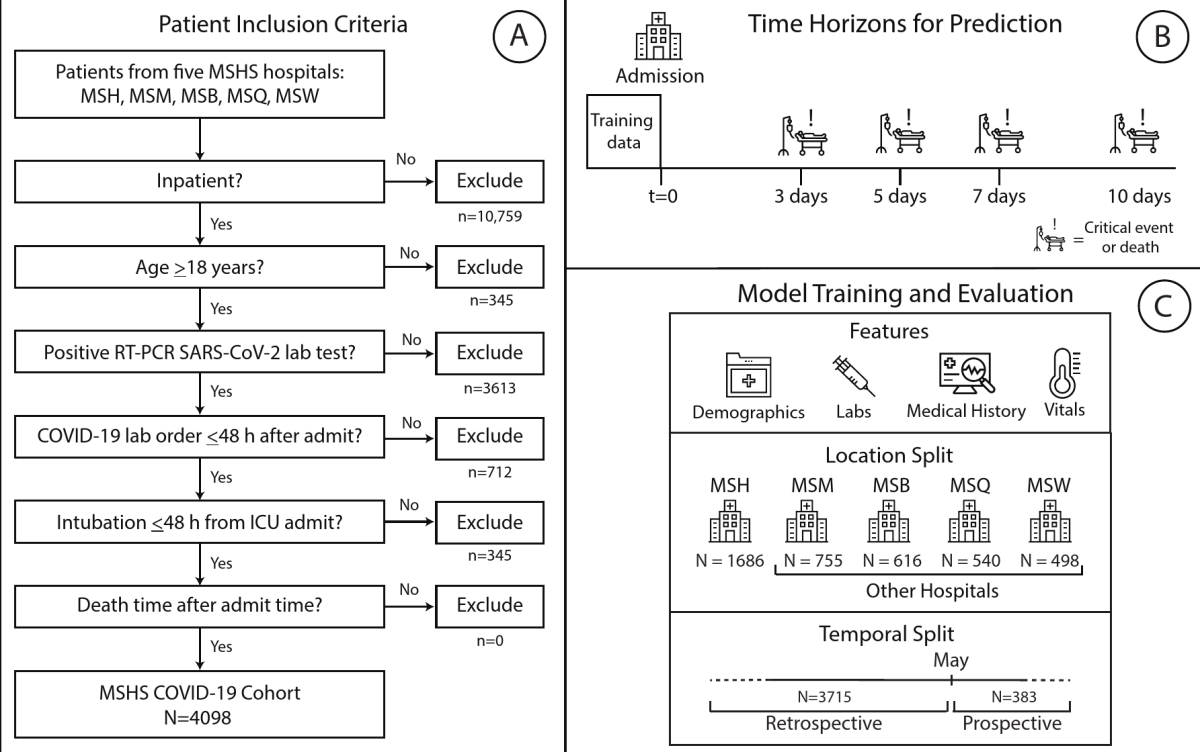
Study design and workflow. (A) Procedure for patient inclusion in our study. (B) Outcomes of interest. We trained the model on data taken at time of admission to predict the likelihood of either mortality or critical event occurrence at 3, 5, 7, and 10 days. (C) Strategy and design of the experiments. Patient clinical data from Mount Sinai Hospital (MSH) before the temporal split (May 1) were used to train and internally validate our XGBoost model in comparison with other baseline models. We then tested the series of XGBoost models on unimputed patient data on patients from four other external hospitals within the MSHS for external validation. h: hours; ICU: intensive care unit; lab: laboratory; MSB: Mount Sinai Brooklyn; MSHS: Mount Sinai Health System; MSM: Mount Sinai Morningside; MSQ: Mount Sinai Queens; MSW: Mount Sinai West (MSW); RT-PCR: reverse transcriptase–polymerase chain reaction; vitals: vital signs.

We retrospectively included all patients who were over 18 years of age, had laboratory-confirmed COVID-19 infection, and were admitted to any of the abovementioned MSHS hospitals between March 15 and May 22, 2020. A confirmed case of COVID-19 was defined by a positive reverse transcriptase–polymerase chain reaction (RT-PCR) assay of a nasopharyngeal swab. To restrict our data to only primary COVID-19–related encounters, we excluded patients who had a first positive COVID-19 RT-PCR result more than two days after admission. We included all patients who had been discharged, had died, or were still admitted and had stayed in the hospital for at least the amount of time corresponding to the outcome in question. This approach provided additional training data for the initial timeframes described in the paper. All exclusion criteria are presented in Figure 1A.

### Study Design

We built predictive models based on data from MSH patients who were admitted from March 15 to May 1, 2020, which was the cutoff time for prospective evaluation. These patients were considered to be part of the internal validation cohort. All patients admitted to other hospitals (OH) were grouped together. To allay concerns about effects of immortal time bias and censoring on the results, we recorded the ultimate outcome of each patient who was admitted in this time frame, even if the outcome occurred after the data enrollment cutoff. For patients within the internal validation cohort, the models were trained and their performance was evaluated through stratified k-fold cross-validation to mitigate the variability of a single train-test split. A final model was then trained for each outcome and time window using all the patients in this data set, and this model was then assessed through a series of validation experiments. First, we externally validated OH patients from March 15 to May 1, 2020, which was the same time frame used to train the model; this afforded benefits by assessing the generalizability of the model to a new setting (Figure 1B). Then, to assess temporal generalizability, we performed prospective validations of the model independently on both MSH and OH patients admitted from May 1 to May 22, 2020 (Figure 1C).

### Study Data

Demographics collected included age, sex, reported race, and ethnicity. Race was collapsed into seven categories based on the most recent US census race categories: American Indian or Alaskan Native, Asian, Black or African American, other, Native Hawaiian or other Pacific Islander, unknown, and White [23]. Ethnicity was collapsed into three categories: Hispanic/Latino, non-Hispanic/Latino, and Unknown.

Additionally, diagnosis codes based on International Classification of Diseases-9/10-Clinical Modification (ICD-9/10-CM) codes and procedures were obtained to identify associated pre-existing conditions. We chose to include conditions with previously reported increased incidence in hospitalized patients with COVID-19: coronary artery disease, heart failure, hypertension, atrial fibrillation, obstructive sleep apnea, asthma, chronic obstructive pulmonary disease, cancer, chronic kidney disease, diabetes, viral hepatitis, liver disease, intracerebral hemorrhage, and stroke [9,24-27]. Inclusion of these chronic conditions and acute kidney injury (AKI) was based on ICD-9/ICD-10 codes related to active problems documented during COVID-19 hospitalization, defined by the presence of at least one ICD code signifying the condition. Laboratory measurements and vital signs near the time of admission were also retrieved for each patient during their hospital encounter. Given the resource constraints due to COVID-19, which delayed acquisition of laboratory results, the first laboratory value in a 36-hour window period was used as the representative laboratory value on admission. The implications of this strategy for the model performance are illustrated in the Multimedia Appendices.

All laboratory orders from the five hospitals were queried for patients included in this study within the timeframe of interest. Due to discrepancies in how laboratory orders were named in different hospitals, a comprehensive and statistical review of all laboratory orders by field name was conducted by a multidisciplinary team of clinicians to ensure direct mapping between all sites. Additionally, many laboratory values represented a single component (eg, sodium) but were acquired from either an arterial blood gas (ABG) test, venous blood gas (VBG) test, or basic metabolic panel (BMP). Based on the utility of these laboratory values in clinical practice and the similarity between their statistical distributions, laboratory values derived from a VBG or BMP were collapsed into a single category (ie, “SODIUM”) and those derived from an ABG were moved to a separate category (ie, “SODIUM_A”). In the set of all laboratory order names that were combined into a single laboratory category, the earliest laboratory result by time was chosen as the representative laboratory value for that category. Finally, laboratory data below the 0.5th percentile and above the 99.5th percentile were removed to avoid inclusion of any obvious outliers that could represent incorrect documentation or measurement error.

### Data Sharing

The raw data used in this work cannot be shared due to patient privacy and security concerns. However, we are open to using this data set for validation of other models through a collaboration under an appropriate data use agreement with the authors at the Icahn School of Medicine at Mount Sinai.

### Definition of Outcomes

The two primary outcomes were (1) death versus survival or discharge and (2) critical illness versus survival or discharge through time horizons of 3, 5, 7, and 10 days. Critical illness was defined as discharge to hospice, intubation ≤48 hours prior to intensive care unit (ICU) admission, ICU admission, or death. A composite outcome (ie, mortality as opposed to discharge or survival) was chosen to bypass issues of competing risks.

### Model Development, Selection, and Experimentation

Our primary model was the Extreme Gradient Boosting (XGBoost) implementation of boosted decision trees on continuous and one-hot encoded categorical features [28]. The XGBoost algorithm provides robust prediction results through an iterative process of prediction summation in decision trees fit to the residual error of the prior ensemble. While each tree is too simple to accurately capture complex phenomena, the combination of many trees in the XGBoost model accommodates nonlinearity and interactions between predictors. The model directs missing values through split points to minimize loss. Hyperparameter tuning was performed by randomized grid searching directed toward maximizing the F1 score metric over 5000 discrete grid options. Ten-fold stratified cross-validation was performed inside each grid option, and the optimal hyperparameter set was chosen based on the model in the grid search with the highest F1 score. Final model hyperparameters for the XGBoost model are listed in Multimedia Appendix 1. To generate confidence intervals for the internal validation set, training and testing was performed for 500 bootstrap iterations with a unique randomly generated seed for the train-test data splits.

We opted to implement our analyses within a classification framework because we aimed to implement our models with regard to clinically relevant time boundaries for resource allocation and clinical decision-making, such as resource allocation, triage, and decisions for ICU transfer. A major goal of our analysis was the construction of a resilient and highly performant predictive model; therefore, the selection of the XGBoost algorithm is reasonable given its well-understood properties as the best-performing machine learning algorithm for classification tasks on tabular data. The XGBoost algorithm also addresses real-life problems such as missing data and highly multidimensional independent variables, while alternate strategies and extensions must be employed to enable Cox proportional hazard analyses in these settings.

To compare the performance of our XGBoost model for the training and internal validation data, we generated two predictive models as a baseline, namely logistic regression (LR) and LR with L1 regularization, given their ubiquity as preferred models in current COVID-19 research. L1 regularization, also known as least absolute shrinkage and selection operator (LASSO), was used to train the LR and impose parsimony in feature selection, given the number of features present in the data set (73). LASSO and LR were optimized by an exhaustive grid search for the inverse regularization parameter (Multimedia Appendix 1). For these baseline models, the issue of missingness was addressed by imputation. Features with >30% missingness were dropped, and k-nearest neighbors (kNN, k=5) was used to impute missing data in the remaining feature space. To further assess the impact of imputation on performance, an XGBoost model was also created and trained on the imputed data set. Imputation for the training set (ie, MSH only) and external validation set (ie, OH) were performed using only the first collected value from the respective sites to prevent information leakage that could compromise assessment of generalizability. We assessed the calibration of the results of each model to ensure that the model predictions could be interpreted as real-world risk scores. Calibration was performed using both the sigmoid and isotonic methods of the *CalibratedClassifierCV* class in scikit-learn and evaluated using the Brier score metric.

### Experimental Evaluation

All models were trained and evaluated using 10-fold stratified cross-validation, and confidence intervals were generated using 500 iterations of bootstrapping. Stratified k-fold cross-validation maintains an outcome distribution across each fold in concordance with the outcome distribution in the study population. We present calibration plots for all these experiments, including isotonic and sigmoid calibrations, that show the proportion of positive cases to the mean predicted value for the raw models in Figures S1-S8 in Multimedia Appendix 2. In these plots and in Multimedia Appendix 3, we also report the Brier score, which measures the quality of calibration (a lower score indicates greater accuracy). Ultimately, we selected the best-calibrated model based on the lowest Brier score, and performed all subsequent experiments with this model. Probability scores output by the model were used to calculate the areas under the receiver operator characteristic curve (AUC-ROCs) and areas under the precision-recall curve (AU-PRCs). The receiver operating characteristic curve shows how the balance between true and false positive rates is affected at different decision thresholds. The precision-recall curve visualizes how the balance of false positives and negatives is affected at different decision thresholds. The decision threshold was calculated separately for each fold to maximize the F1 score for prediction of the primary outcome. The threshold for the final model was taken as the median of the calculated thresholds across the 10 cross-validation folds. Accuracy, F1 score, sensitivity, and specificity were calculated on the basis of these thresholds. Model performance was assessed during internal cross-validation, external validation, and prospective validation. The models were compared on the basis of their AUC-ROC and AU-PRC values across the time intervals in each population of patients. The AU-PRC is known to be a better metric in skewed data sets that have greater class imbalance and was therefore primarily used in the model evaluation and selection.

### Model Interpretation

We evaluated feature contributions toward model prediction using SHAP scores. SHAP scores are a game-theoretic approach to model interpretability; they provide explanations of global model structures based upon combinations of several local explanations for each prediction [29]. To interpret and rank the significance of input features toward the final prediction of the model, mean absolute SHAP values were calculated for each feature across all observations in the internal validation set. We also plotted a heatmap showing SHAP interaction values, which are an extension of SHAP summary values to capture how pairwise interactions between different features contribute to model prediction. For a given pair of features, their pairwise interaction effect is calculated after removing the individual effects of those features. Values on the diagonal represent the main effects (ie, the SHAP summary values), and values off the diagonal represent the interaction effects. Higher values on the heatmap (ie, brighter squares) represent a greater impact on model predictions. In addition, we calculated the feature importance from the coefficients of the LASSO model (Figure S9 in Multimedia Appendix 2).

### Promoting Interoperability and Replicability

This article is written following the TRIPOD (Transparent Reporting of a Multivariable Prediction Model for Individual Prognosis or Diagnosis) guidelines [30], which are further elaborated in Multimedia Appendix 4. Furthermore, we release all code used to build the classifier under the GPLv3 license in a public GitHub repository [31].

## Results

### Clinical Data Source and Study Population

Electronic health records for 4098 COVID-19–positive inpatient admissions at five hospitals within the MSHS between March 15 and May 22, 2020, were retrieved for data analysis based on the inclusion criteria. These data included patient demographics, past medical history, and admission vital signs and laboratory test results (Table 1 and Table 2; Multimedia Appendix 5). Vital sign and laboratory test data were included as baseline features in order to work within the bounds of the processing and operations involved in obtaining the results of these tests. No data leakage occurred, and we did not find disproportionate rates of feature missingness for patients who died within this time window for feature inclusion (see the Multimedia Appendices). We show the number of patients involved and the proportion of events in each experiment by time window in Multimedia Appendix 6. Relevant patient events (intubation, discharge to hospice care, or death) were recorded, and subsets were constructed at 3-, 5-, 7-, and 10-day intervals after admission (Figure 1). Before May 1, 21.3% to 35.3% of patients had experienced a critical event (intubation, ICU admission, discharge to hospice care, or death) across all time intervals. On or after May 1, this proportion changed to 14.3% to 21.9%. Similarly, before May 1, 2.6% to 22.4% patients died across all time intervals, with the proportion changing to 1.1% to 8.0% on or after May 1. The survival curve for mortality is shown in Figure S10 in Multimedia Appendix 2. This curve was generated by fitting a Kaplan-Meier estimator to the survival time for patients with observed (in-hospital) death instead of discharge (Multimedia Appendix 6). In contrast, the set of noncases consisted of patients with all other discharge dispositions and those who were still hospitalized at the respective intervals after admission.

**Table 1:** Demographic characteristics, clinical history, and vital signs of hospitalized patients with COVID-19 at baseline (N=4098).

Characteristic on admission				Retrospective			Prospective		
			MSH^a^ (n=1514)		OH^b^ (n=2201)		MSH		OH
**Demographics**									
	**Sex, n (%)**								
		Male	869 (57.4)		1257 (57.1)		104 (59.4)		104 (50)
		Female	645 (42.6)		944 (42.9)		71 (40.6)		104 (50)
	**Race, n (%)**								
		Other	639 (42.2)		804 (36.5)		80 (45.7)		53 (25.5)
		Caucasian	354 (23.4)		533 (24.2)		43 (24.6)		56 (26.9)
		African American	357 (23.6)		688 (31.3)		37 (21.1)		79 (38)
		Unknown	80 (5.3)		45 (2)		—^c^		—
		Asian	77 (5.1)		102 (4.6)		10 (5.7)		11 (5.3)
		Pacific Islander	—		—		—		—
	**Ethnicity, n (%)**								
		Non-Hispanic/Latino	820 (54.2)		1377 (62.6)		98 (56)		139 (66.8)
		Hispanic/Latino	421 (27.8)		556 (25.3)		50 (28.6)		43 (20.7)
		Unknown	271 (17.9)		236 (10.7)		24 (13.7)		26 (12.5)
	Age, median (IQR)		62.9 (50.7-73)		69.6 (53.3-80)		63.7 (51.2-73.8)		69.8 (55.5-79.9)
	**Age (years), n (%)**								
		18-30	64 (4.2)		46 (2.1)		16 (9.1)		—
		31-40	155 (10.2)		113 (5.1)		13 (7.4)		12 (5.8)
		41-50	165 (10.9)		160 (7.3)		14 (8)		17 (8.2)
		51-60	291 (19.2)		341 (15.5)		33 (18.9)		35 (16.8)
		61-70	394 (30)		517 (20)		40 (20)		39 (20)
		71-80	258 (17)		522 (23.7)		41 (23.4)		52 (25)
		81-90	142 (9.4)		396 (18)		13 (7.4)		38 (18.3)
		≥90	45 (3)		106 (5)		—		—
**Previous medical history, n (%)**									
	Hypertension		64 (4.2)		46 (2.1)		63 (40)		83 (40)
	Atrial fibrillation		155 (10.2)		113 (5.1)		13 (7)		21 (10)
	Coronary artery disease		165 (10.9)		160 (7.3)		32 (20)		41 (20)
	Heart failure		291 (19.2)		341 (15.5)		26 (10)		30 (10)
	Stroke		394 (30)		517 (20)		16 (9)		10 (5)
	Chronic kidney disease		258 (17)		522 (23.7)		32 (20)		43 (20)
	Diabetes		142 (9.4)		396 (18)		40 (20)		54 (30)
	Asthma		45 (3)		106 (5)		11 (6)		—
	Chronic obstructive pulmonary disease		64 (4.2)		46 (2.1)		13 (7)		11 (5)
	Cancer		158 (10)		124 (6)		43 (20)		14 (7)
**Vital signs at hospital admission, median (IQR)**									
	Heart rate (beats per minute)		87 (77-97)		86 (76-98)		85 (74-97.5)		82 (72.8-96)
	Pulse oximetry (%)		96 (94-97)		96 (94-98)		97 (95-98)		97 (96-98)
	Respiration Rate (breaths per minute)		20 (18-21)		18 (18-20)		18 (18-20)		18 (18-20)
	Temperature (ºF)		98.7 (98-99.9)		98.5 (97.7- 99.3)		98.1 (97.5-98.6)		97.9 (97.3-98.6)
	Systolic blood pressure (mm Hg)		125 (112-140)		125 (111-140)		122 (111.5-138)		127 (112.8-141.2)
	Diastolic blood pressure (mm Hg)		69 (61-78)		72 (64-80)		70 (60.5-78.5)		72 (64-82)
	BMI (kg/m^2^)		28.1 (24.4-32.8)		27.5 (24.2-32.5)		25.92 (21.9-30.4)		27.7 (23.4-32.1)

^a^MSH: Mount Sinai Hospital.

^b^OH: other hospitals.

^c^—: Values with fewer than 10 patients per field are censored to protect patient privacy.

**Table 2:** Admission laboratory parameters of hospitalized patients with COVID-19 at baseline (N=4098), median (IQR).

Laboratory parameters		Retrospective			Prospective	
		MSH^a^ (n=1514)	OH^b^ (n=2201)	MSH		OH
**Metabolic markers**						
	Sodium (mEq/L)	138 (135-140)	139 (136-142)	139 (136-141)		139 (136-141)
	Potassium (mEq/L)	4 (3.6-4.5)	4.3 (3.9-4.7)	4 (3.7-4.4)		4.3 (3.8-4.6)
	Creatinine (mg/dL)	0.91 (0.7-1.5)	1.01 (0.8-1.7)	0.89 (0.7-1.6)		1.12 (0.7-2.1)
	Lactate (mg/dL)	1.8 (1.4-2.3)	1.4 (1.1-2)	1.8 (1.4-2.3)		1.49 (1-1.9)
**Hematological markers**						
	White blood cells (10^3^/µL)	7 (5-10.2)	7.6 (5.5-10.9)	7.3 (5.1-10.7)		8.3 (6.3-11.9)
	Lymphocyte percentage	NA (NA-NA)	14.2 (8.6-21.3)	NA (NA-NA)		14.7 (9.9-21.6)
	Hemoglobin (mEq/L)	12.2 (10.7-13.5)	12.7 (11.1-13.9)	10.5 (9.1-12.8)		11.1 (9.2-12.8)
	Red blood cell distribution width (%)	4.2 (3.7-4.6)	4.28 (3.8-4.7)	3.69 (3.1-4.3)		3.79 (3.2-4.5)
	Platelets (n)	220 (165-291)	208 (158-281)	224 (166.2-304)		211 (149.2-285.2)
**Liver function**						
	Alanine aminotransferase (units/L)	30 (18-53)	31 (19-54)	26 (13.8-51)		23 (14-36)
	Aspartate aminotransferase (units/L)	42 (28-66)	45 (30-74)	30 (20-50.5)		30 (19-49)
	Albumin (g/dL)	2.9 (2.5-3.2)	2.9 (2.5-3.2)	2.9 (2.5-3.4)		2.9 (2.3-3.3)
	Total bilirubin (mg/dL)	0.6 (0.4-0.8)	0.6 (0.4-0.8)	0.7 (0.4-1)		0.5 (0.4-0.7)
**Coagulation markers**						
	Prothrombin time (s)	14.5 (13.6-16)	14.9 (13.9-16.5)	14.8 (13.6-16.2)		15.05 (13.7-17.6)
	Partial Thromboplastin time (s)	32.9 (29.2-38.5)	34.8 (30.3-41.5)	32.6 (28.8-37.8)		36.1 (31-45.9)
**Gases**						
	PCO_2_^c^ (mmHg)	42 (37-47)	42 (37-53)	44 (39-49)		42 (37-48.5)
	pH	7.4 (7.3-7.4)	7.36 (7.3-7.4)	7.39 (7.4-7.4)		7.36 (7.3-7.4)
**Inflammatory markers**						
	C-reactive protein (mg/L)	116.4 (57.1-199.5)	132.4 (65.8-218.9)	62.2 (17-148.9)		73.7 (33.5-181.8)
	Ferritin (ng/mL)	800 (365-1916)	906 (438-2056)	485 (200.2-1031.5)		690 (303.5-1470.2)
	D-dimer (ng/mL)	1.44 (0.8-3)	2.42 (1.2-4.4)	1.66 (0.9-3.1)		1.97 (1.1-3.8)
	Creatinine phosphokinase (units/L)	146 (70-488)	220 (76.8-501.8)	194.5 (93.2-290.8)		271.5 (48.8-611.5)
	Lactate dehydrogenase (units/L)	423 (315-571)	466.5 (356.2-652.2)	334 (251.5-472)		364 (266.8-487)
**Cardiac markers**						
	Troponin I (ng/mL)	0.05 (0-0.2)	0.064 (0-0.2)	0.05 (0-0.1)		0.0525 (0-0.1)

^a^MSH: Mount Sinai Hospital.

^b^OH: other hospitals.

^c^PCO_2_: partial pressure of carbon dioxide.

### Classifier Training and Performance

**Figure 2:**
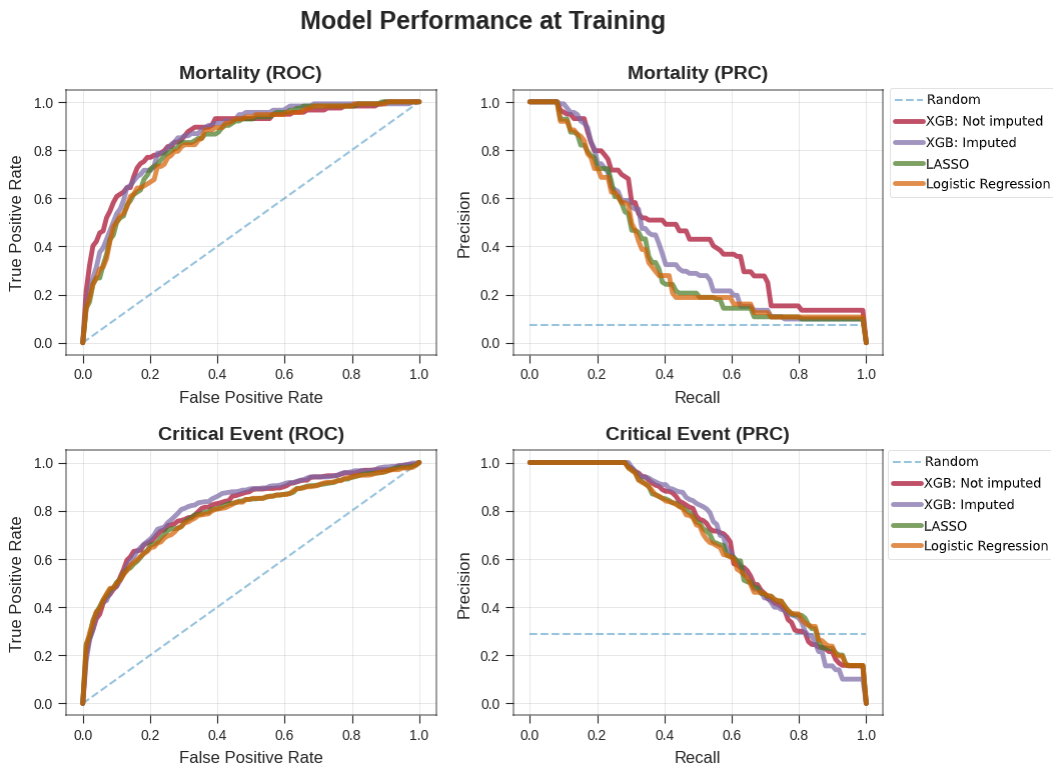
Comparison of the performance of the XGBoost and baseline models. Performance of the XGBoost classifier by ROC curves (left) and PR curves (right) on the unimputed data set (red) for mortality (top) and critical event (bottom) prediction versus the three baseline models: XGBoost classifier on the imputed data set (purple), LASSO (green), and LR (orange). LASSO: least absolute shrinkage and selection operator; PRC: precision-recall curve; ROC: receiver operating characteristic; XGB: Extreme Gradient Boosting.

We developed models based on cross-validation experiments for all model types and conditions within the MSH at the earlier time period of the study (ie, up to the enrollment date cutoff). On internal cross-validation for mortality prediction, the unimputed XGBoost model demonstrated strong performance, with AUC-ROC values ranging from 0.84 to 0.90 and AU-PRC values ranging from 0.33 to 0.48 (Multimedia Appendix 6). In comparison, LR and LASSO, after kNN imputation on the missing data elements, performed marginally worse on every outcome, with AUC-ROC values ranging from 0.80 to 0.82 and AU-PRC values ranging from 0.10 to 0.40 (Figure 2, Multimedia Appendix 6). Additionally, when trained with imputed data, the XGBoost classifier performed worse, achieving AUC-ROC values from 0.80 to 0.84 and AU-PRC values from 0.18 to 0.40 across all time periods. In the case of internal validation for critical event prediction, the AUC-ROC values of the unimputed XGBoost model ranged from 0.79 to 0.81, and the AU-PRC values ranged from 0.60 to 0.70. The performance for the LASSO and LR models with imputation was poorer, with AUC-ROC values of 0.75 to 0.77 and AU-PRC values of 0.54 to 0.65.

We then assessed the performance of these models in three validation experiments: in OH within the same time period, within the same hospital (MSH) at a future time period, and in OH at a future time period. The unimputed XGBoost AUC-ROC generally showed the best performance for mortality prediction across intervals ranging from 0.84 to 0.88, with AU-PRC values ranging from 0.44 to 0.64. For LR and LASSO, the AUC-ROC values ranged from 0.82 to 0.83, while the AU-PRC values ranged from 0.22 to 0.58. The imputed XGBoost continued to perform slightly worse, with AUC-ROC values ranging from 0.72 to 0.83 and AU-PRC values ranging from 0.17 to 0.60. For critical event prediction across all time intervals, the AUC-ROC values of the unimputed XGBoost model ranged from 0.78 to 0.81, while the AU-PRC values ranged from 0.51 to 0.69. Performance of LR and LASSO was marginally worse, with ranges of 0.74 to 0.81 for the AUC-ROC and 0.44 to 0.70 for the AU-PRC. The performance of imputed XGBoost was similar to that of unimputed XGBoost, with AUC-ROCs ranging from 0.76 to 0.82 and AU-PRCs ranging from 0.49 to 0.71.

Similarly, in prospective validation at OH for mortality prediction across all time intervals, the AUC-ROC values of the unimputed XGBoost model ranged from 0.68 to 0.88, while the AU-PRC values ranged from 0.13 to 0.31. The performance of the LR and LASSO models in the same experiments was much poorer in terms of AUC-ROC, with values ranging from 0.51 to 0.74, and at par with the unimputed XGBoost model in terms of AU-PRC, with values ranging from 0.13 to 0.34. Imputed XGBoost performed worse overall, with AUC-ROC values ranging from 0.66 to 0.81 and AU-PRC values between 0.06 and 0.21. In the case of prospective validation for critical event prediction at OH, the AUC-ROC values of the unimputed XGBoost model ranged from 0.74 to 0.77, and the AU-PRC values were between 0.36 and 0.50. In contrast, the performance of the LR and LASSO models over the same conditions was poorer overall, with ranges of 0.65 to 0.74 for the AUC-ROC and 0.31 to 0.46 for the AU-PRC. The imputed XGBoost model again performed slightly worse than the unimputed XGBoost model, with AUC-ROC values from 0.71 to 0.77 and AU-PRC values between 0.31 and 0.48. Prospective validation at MSH presented a new set of challenges for all the models because of the generally lower number of outcomes and larger class imbalance for mortality prediction for the shorter time intervals. For mortality prediction overall, the AUC-ROC values of the unimputed XGBoost model ranged from 0.85 to 0.96, and the AU-PRC values ranged from 0.32 to 0.55. The LR and LASSO models showed much poorer performance, with AUC-ROC values ranging from 0.44 to 0.85 and AU-PRC values ranging from 0.01 to 0.41. The imputed XGBoost model also performed worse than the unimputed XGBoost model, with AUC-ROC values of 0.82 to 0.88 and AU-PRC values of 0.04 to 0.50. For prediction of critical events, the AUC-ROC values of the unimputed XGBoost model were between 0.72 and 0.78, and its AU-PRC values were between 0.40 and 0.54. The performance of the LR and LASSO models in the same set of experiments was slightly poorer, with ranges of 0.66 to 0.75 for the AUC-ROC and 0.32 to 0.48 for the AU-PRC. The imputed XGBoost model performed marginally worse than the unimputed XGBoost model, with values of 0.71 to 0.77 for the AUC-ROC and 0.42 to 0.50 for the AU-PRC.

Model calibration as measured by Brier scores improved after either sigmoid or isotonic calibration across all time windows. For the unimputed XGBoost model, isotonic calibration performed better than sigmoid calibration, with Brier scores ranging from 0.124 to 0.161 for critical event prediction and from 0.019 to 0.085 for mortality prediction. Sigmoid calibration only slightly outperformed isotonic calibration for critical event prediction at 10 days (Brier scores of 0.160 vs 0.161, respectively).

### Model Feature Importance

**Figure 3:**
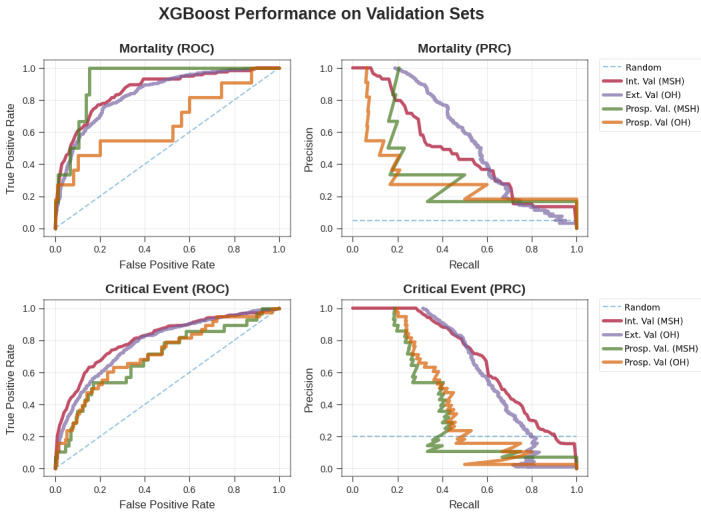
Performance of the XGBoost classifier by ROC curves (left) and precision-recall curves (right) for mortality (top) and critical events (bottom) in validation experiments of generalizability and time. For generalizability, we show our XGBoost model from cross-validation on MSH and applied to all other hospitals. We also show the performance of the model on prospective patients who were unseen at the time of the original experiment at MSH and all other hospitals in the same time frame. Ext. Val.: external validation; Int. Val.: internal validation; MSH: Mount Sinai Hospital; OH: other hospitals; PRC: precision-recall curve; Prosp. Val: prospective validation; ROC: receiver operating characteristic.

Mean absolute SHAP values [32] were calculated for each XGBoost model in the internal validation data set (Figure 3). For critical event prediction, the presence of acute kidney injury and both high and low levels of lactate dehydrogenase (LDH), respiratory rate, and glucose were strong drivers for predicting a critical event within one week. Other notable drivers of predictability included both systolic and diastolic blood pressure, pH, total protein levels, C-reactive protein, and D-dimer. For mortality, both high and low values for age, anion gap, C-reactive protein, and LDH were the strongest effectors in guiding mortality prediction within one week of admission. Other important variables for increasing the prediction of death included oxygen saturation on intake admission, blood urea nitrogen, ferritin, red cell distribution width (RDW), diastolic blood pressure, and lactate. Finally, using SHAP interaction scores, we discovered that covariate interactions between features contributed less to the predictions of the models than the independent importance of each feature (Figures S11 and S12 in Multimedia Appendix 2), except for the case of AKI, where levels of LDH, glucose, and C-reactive protein were strong covariates. As a comparison, we also assessed the feature importance for the LASSO model for these experiments (Figure S9 in Multimedia Appendix 2). We saw an overlap of key features that both models considered important in their predictions for both critical event and mortality prediction at 7 days. For critical events, we found that AKI was the most important feature in both models. Higher respirations and D-dimer levels were also associated with higher mortality, and lower diastolic blood pressure was negatively associated. For mortality, we also saw strong concordance in key features between both models. Specifically, older age and higher anion gap were strong contributors to mortality prediction in both models, and lower diastolic blood pressure and oxygen saturation were negatively associated with mortality. It is encouraging that many of the features with high importance in the primary XGBoost model were also prioritized in the LASSO classifier, suggesting the robustness of the predictive ability of these features. The top 10 features for the Critical Event and Mortality models at seven days are enumerated in Multimedia Appendix 7**.**

## Discussion

### Principal Findings

In this work, we performed a series of experiments with the goal of using machine learning to predict in-hospital mortality or critical events from admission for patients with COVID-19. We highlight several important findings with implications for clinical medicine. First, we offer a robust prediction algorithm pertaining to the most clinically severe outcomes based solely on admission metrics, which maintains its training performance in both external and prospective validation experiments. Most notably, the high specificity in predicting mortality within 3, 5, and 7 days of admission (AU-PRCs of 0.91 to 0.97) suggests a role of the algorithm in augmenting clinicians’ decision-making when identifying patients at immediate risk of impending clinical decompensation and potential in guiding allocation of more intensive care upon admission. Finally, the impact of the large class imbalance and missingness on model training and performance can be appreciated when comparing mortality predictions at 3 days. On the non-imputed data set, the XGBoost classifier achieves a remarkably higher AU-PRC (0.44) compared to the models using imputed data (0.14 for LR and LASSO, 0.12 for XGBoost imputed). It is important to note the consideration of the AU-PRC instead of the AUC-ROC for deriving this claim, as the AU-PRC includes both precision (ie, positive predictive value) and recall (ie, sensitivity) and thus accounts for the class imbalance, which the AUC-ROC metric generally ignores. Overall, we found that the unimputed XGBoost model performed better not only in internal validation but in the vast majority of the other validation experiments. As such, we believe it can be generalized more readily than the other models to new cohorts and time points. Along these lines, we found that our imputation strategy generally hindered the performance of the XGBoost model. There were instances where the XGBoost model performed approximately the same (within the bounds of the confidence intervals) or worse than the other comparators for different metrics. For instance, in the prospective OH experiment for predicting critical events within 7 and 10 days, the LASSO method outperformed the unimputed XGBoost model in terms of AUC-ROC and AU-PRC. In the 7-day condition, however, the imputed XGBoost model actually performed the best overall, which suggests that the imputation strategy worked better in this particular scenario. Additionally, in the prospective OH experiment, the unimputed XGBoost model underperformed compared to the other models for mortality prediction; however, we believe this was due to the extremely low positive prevalence. Thus, while XGBoost makes assumptions on how it handles missing data, we found that XGBoost without imputation was the more robust method in these experiments. Furthermore, this strategy is conducive to implementation into clinical operations, as it removes the need for an intermediary imputation step.

**Figure 4:**
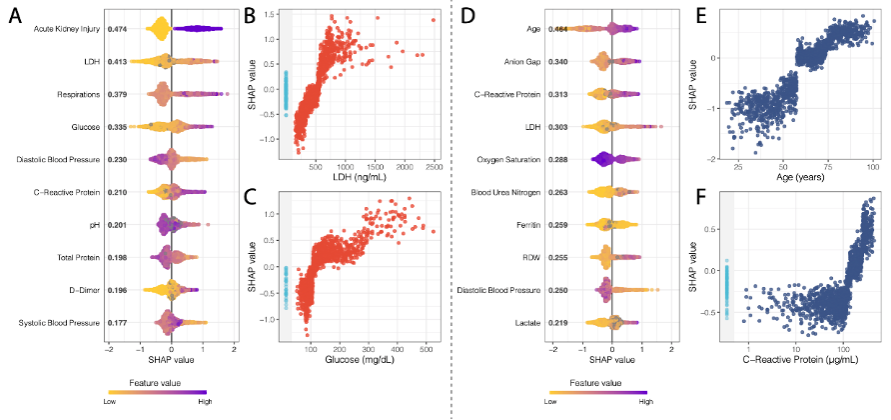
SHAP summary plots for critical event (A) and mortality (D) at 7 days showing the SHAP values for the 10 most important features for the respective XGBoost models. Features in the summary plots (y-axis) are organized by their mean absolute SHAP values (x-axis), which represent the importance of the features in driving the prediction of the classifiers for patients. (B) and (C) Dependency plots demonstrating how different values can affect the SHAP score and ultimately impact classifier decisions for LDH and glucose, respectively, for critical event prediction. (E) and (F) Dependency plots for age and C-reactive protein levels. Patients with missing values for a feature in the dependency plot are clustered in the shaded area to the left. LDH: lactate dehydrogenase; RDW: red cell distribution width; SHAP: SHapley Additive exPlanation.

Additionally, our framework permits a clinically relevant understanding of the most salient features of the unimputed XGBoost model, defining its decision boundaries using patients from the holdout set during internal validation (Figure 4). At 7 days, age was the most important feature for mortality prediction in COVID-19–positive patients, with a notably rapid and nonlinear increase of feature contribution with increasing age (Figure 4) [33,34]. Hyperglycemia, particularly in the ranges that catered to positive predictions (Figure 4C), may serve as proxies for either metabolic syndrome, diabetic ketoacidosis, or hyperosmolar hyperglycemic state predisposition from underlying diabetes, which have previously also been reported and associated with poorer outcomes in COVID-19–positive patients [35-37]. The higher information content in continuous values such as glucose levels and their larger role in the level of control of diabetes is a likely explanation for why diabetes, as a comorbidity, failed to be a strong driver of prediction. The demonstration of the anion gap, in conjunction with high levels of lactate, as another strong model influencer for mortality prediction is likely linked with potential ongoing elevated anion-gap metabolic acidosis from a brewing severe inflammatory response syndrome or sepsis picture [38]. Elevation in serum LDH is a nonspecific marker of inflammation; however, it is implicated in pulmonary endothelial cell injury and in COVID-19–positive patients [39-41]. AKI has been reported in patients with severe COVID-19 and, if present early, may be a strong indicator of future critical events [42,43]. The covariate relationship between LDH, CRP, and glucose may reflect underlying severe inflammation and deranged metabolism, which may be contributing to the AKI. Elevated RDW, which may be an index of enhanced patient frailty and risk of adverse outcomes [35], was also a strong driver of mortality. Additionally, vital sign instability (low oxygen saturation, tachypnea, hypotension), elevated ferritin [41,44], high lactate, and acidosis were contributors to driving model predictions toward mortality. With growing evidence of COVID-19–induced hypercoagulable states in these patients [41,45,46], it is promising that our model recognized the feature importance of coagulability markers such as D-dimer (Figure 4). Thus, this corroboration of the features learned by XGBoost and highlighted by the SHAP analysis with the findings from pathophysiological principles and more recent correlative studies exploring patients with COVID-19 [2,3,9,25,26,47,48] gives additional credibility to these findings. Additionally, when we compared these features to those that were ranked highly for the LASSO model, we found many concordant features with the same direction of effect; this further strengthens the evidence of the utility of these features in predictive models (Figure S9 in Multimedia Appendix 2).

Just as interesting as the most important features identified for classification by XGBoost are the features that were not prioritized (ie, much lower mean absolute SHAP values). For example, race is a social construct that at best serves as a proxy for the social disparities leading to infection risk at the population level, and it is also related to the distribution of comorbid conditions that potentiate disease severity. Furthermore, race is both poorly represented (including a category for “Unknown”) and inadequately characterized in the EHR. While race, in and of itself, potentially carries a large amount of information because it inadvertently represents the very societal inequities that lead to poorer outcomes (ie, structural racism as a contributor to COVID-19 health disparities), the model instead chose to prioritize more objective markers of health status (laboratory values, vital signs, comorbidities) that more directly represent the deeper biology of the risk factors and state of disease severity leading to these adverse outcomes. Contrary to our expectation, age was not identified as a significant feature for critical event prediction within 7 days in the primary analyses. This suggests that the model decided to capture acute critical events by relying on more objective measures that are not confounded by other factors that are cached into age, which may better represent illness severity and more irreversible outcomes (ie, death). Age may then be a better marker for mortality by offering a more stable container of clinical information, given its invariance to change relative to other features.

### Limitations

The results of our models should be considered in light of several limitations. First, we based our predictions solely on data extracted around patient admission (ie, within 36 hours). This step was added purposefully to remove potential bias from effects of hospital workflow, and we found that it did not cause another source bias relating to informed missingness (see Multimedia Appendices). No information from the future was leaked into this prediction. Although the restriction of using data at admission encourages the use of this model in patient triage, events during a patient’s hospital stay after admission may drive their clinical course away from the prior probability, which cannot be captured by baseline admission features. We believe a “live” or continuously updating modelling approach would be better suited for this as a future direction. Furthermore, not all patient laboratory values are drawn at admission, which introduces an element of missingness in our data set. For example, unlike the general patient population, patients on anticoagulation therapy, who likely have comorbidities increasing their baseline risk, will have coagulation laboratory tests (prothrombin time, partial thromboplastin time) performed on admission. We attempted to mediate this issue by including a missingness threshold cutoff, assessing model performance with imputation, and not including any laboratory test that was specific to an intervention (ie, arterial laboratory tests performed in the ICU). Additionally, patients admitted to the hospital later in the crisis benefited from improved patient care protocols from experiential learning but were also negatively affected by resource constraints from overburdened hospitals. These effects may also induce temporal variation between patient outcomes, which is demonstrated by the lower critical event and mortality rate in the prospective validation data set. However, determining the models’ performance in this scenario was one of the justifications for including a future time point. Despite a certain dip in overall performance for the unimputed XGBoost model, which we attribute to heavy imbalance of outcomes and extremely low prevalence rates, we were overall encouraged by its performance. Furthermore, inherent limitations exist when using EHRs, especially those integrated from multiple hospitals. To facilitate timely dissemination of our results, we chose not to manually chart review patient notes that may have otherwise provided additional potential features, such as symptoms and clinical course, to incorporate in our model. Because all five hospitals operate in a single health system, system-wide protocols in laboratory order sets and management protocols were an additional source of bias that may lower external validity. Other interhospital effects, such as shuttling COVID-19 cases to certain hospitals to balance system-wide patient burden, may also imbalance case severity across hospitals and care management between hospitals. This was ultimately a major reason to restrict the model training to a single center and perform testing in other hospital centers. Additionally, in this paper, we present outcome classification derived from a learned optimization threshold cutoff. Further work is needed to identify clinically relevant thresholds for classifying predicted probabilities. Finally, although XGBoost is superior to other models in handling missing data, a notable drawback is its bias toward continuous features instead of categorical ones [49]. However, collinearities between some categorical features in this data set may be present with other continuous features, as exhibited by the covariance strength between hypertension and systolic blood pressure and creatinine in Figure S1 in Multimedia Appendix 2, which can then serve as vehicles for capturing these categorical pieces of information.

### Conclusions

The COVID-19 pandemic unequivocally represents an unprecedented public health crisis. Health care institutions are facing extreme difficulties in managing resources and personnel. Physicians are treating record numbers of patients and are continuously exposing themselves to a highly contagious and virulent disease with varying symptomatology. Only a few therapeutic options have demonstrated improvement in patient outcomes. Our externally and prospectively validated models successfully predict critical illness and mortality up to 10 days in advance in a diverse patient population from admission information alone. We believe that this model also identified important markers for acute care prognosis that can be used by health care institutions to improve care decisions at both the physician and hospital level for management of COVID-19–positive patients.

## Appendix

Multimedia Appendix 1 Supplementary Table 1: Final XGBoost, LASSO and logistic regression model hyperparameters.

Multimedia Appendix 2 Supplementary figures.

Multimedia Appendix 3 Supplementary Table 2: Brier scores for each model and calibration type.

Multimedia Appendix 4 Supplementary Table 3: TRIPOD guidelines.

Multimedia Appendix 5 Supplementary Table 4: Baseline feature variability across all patients.

Multimedia Appendix 6 Supplementary Table 5: Model performance by experiment.

Multimedia Appendix 7 Supplementary Table 6: Clinical features ranked in decreasing order based on their significance (most significant predictor=1, least significant predictor=10) for critical event and mortality at 7 days.

## Abbreviations

ABG: arterial blood gas
AKI: acute kidney injury
AUC-ROC: area under the receiving operator characteristic curve
AU-PRC: area under the precision-recall curve
BMP: basic metabolic panel
EHR: electronic health record
ICU: intensive care unit
ICD-9/10-CM: International Classification of Diseases-9/10-Clinical Modification
kNN: k-nearest neighbors
LASSO: least absolute shrinkage and selection operator
LDH: lactate dehydrogenase
LR: logistic regression
MSB: Mount Sinai Brooklyn
MSH: Mount Sinai Hospital
MSHS: Mount Sinai Health System
MSM: Mount Sinai Morningside
MSQ: Mount Sinai Queens
MSW: Mount Sinai West
OH: other hospitals
RDW: red cell distribution width
RT-PCR: reverse transcriptase–polymerase chain reaction
SHAP: SHapley Additive exPlanation
TRIPOD: Transparent Reporting of a Multivariable Prediction Model for Individual Prognosis or Diagnosis
VBG: venous blood gas
XGBoost: Extreme Gradient Boosting
