# Supplementary material for: Machine Learning to Predict Mortality and Critical Events in a Cohort of Patients With COVID-19 in New York City: Model Development and Validation
Source: J Med Internet Res. 2020 Nov 6;22(11):e24018. doi: 10.2196/24018 (PMC7652593; doi:10.2196/24018)
Supplement: Multimedia Appendix 1 [file jmir_v22i11e24018_app1.docx]

**Supplementary Table 1. Final XGBoost, LASSO and Logistic Regression Model Hyperparameters.**

| **Hyperparameter** | **Critical outcome** | | | | **Mortality** | | | |
| --- | --- | --- | --- | --- | --- | --- | --- | --- |
|  | *3 Days* | *5 Days* | *7 Days* | *10 Days* | *3 Days* | *5 Days* | *7 Days* | *10 Days* |
| **XGBoost** | | | | | | | | |
| reg_alpha | 1.0 | 0.10 | 0.01 | 0.1 | 1.0 | 1.0 | 0.1 | 0.1 |
| min_child_weight | 5.0 | 3.0 | 3.0 | 7.0 | 5.0 | 1.0 | 1.0 | 5.0 |
| max_depth | 9.0 | 6.0 | 6.0 | 6.0 | 6.0 | 9.0 | 6.0 | 6.0 |
| learning_rate | 0.05 | 0.05 | 0.05 | 0.05 | 0.05 | 0.05 | 0.05 | 0.1 |
| gamma | 0.4 | 0.3 | 0.2 | 0.1 | 0.3 | 0.2 | 0.3 | 0.4 |
| colsample_bytree | 0.3 | 0.3 | 0.4 | 0.3 | 0.3 | 0.3 | 0.3 | 0.5 |
| **C:** | | | | | | | | |
| LASSO | 0.01 | 0.01 | 0.01 | 10 | 0.001 | 1 | 0.01 | 0.001 |
| Logistic Regression | 100 | 1 | 10 | 100 | 0.1 | 10 | 10 | 1 |
