# Supplementary material for: Machine Learning to Predict Mortality and Critical Events in a Cohort of Patients With COVID-19 in New York City: Model Development and Validation
Source: J Med Internet Res. 2020 Nov 6;22(11):e24018. doi: 10.2196/24018 (PMC7652593; doi:10.2196/24018)

# Supplementary Figures

Figure S1: XGBoost unimputed calibration plots predicting a critical event at 3, 5, 7, and 10 days.


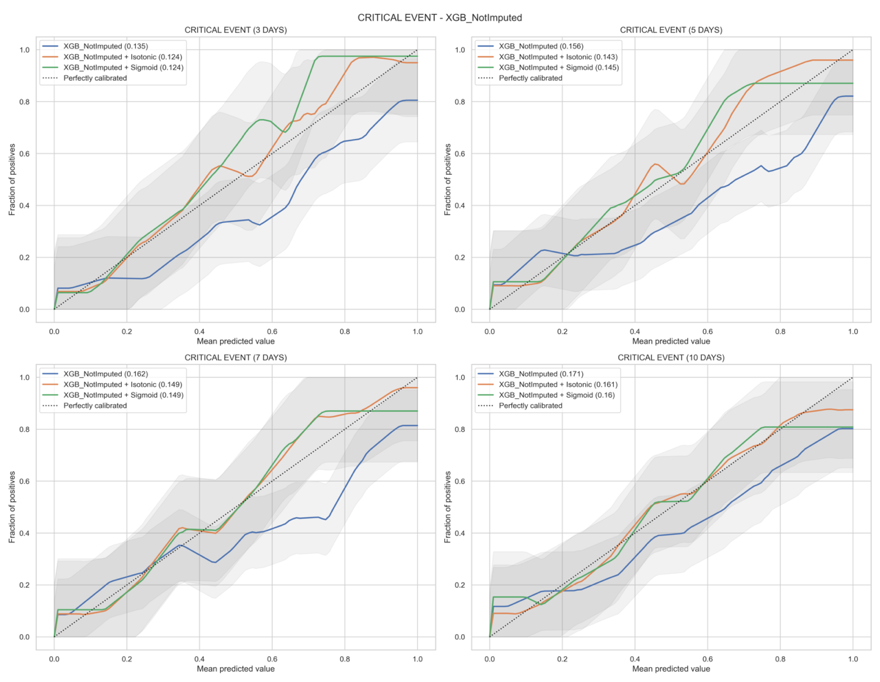


Figure S2: XGBoost unimputed calibration plots predicting mortality at 3, 5, 7, and 10 days.


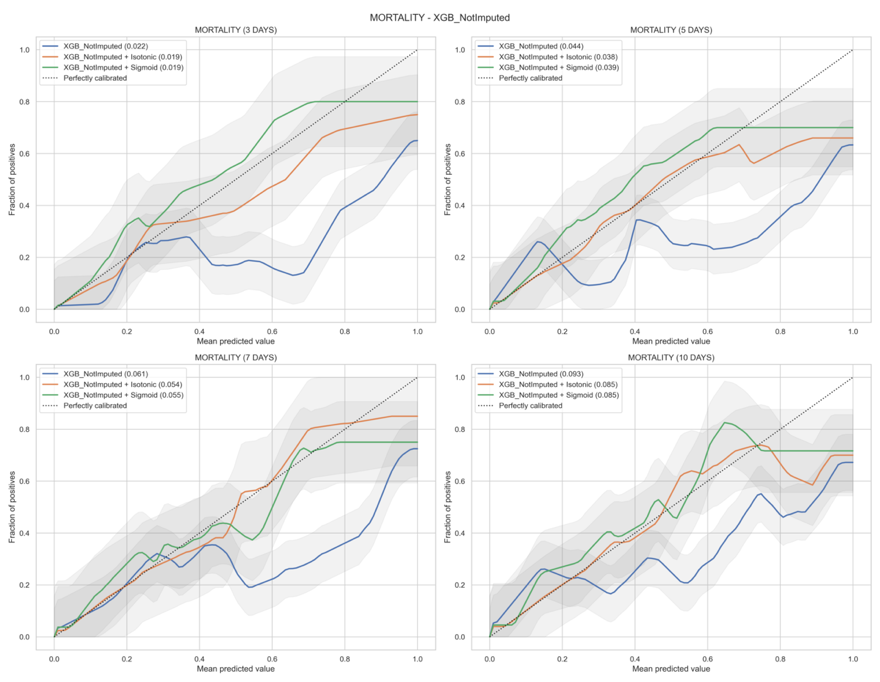


Figure S3: XGBoost imputed calibration plots predicting a critical event at 3, 5, 7, and 10 days.


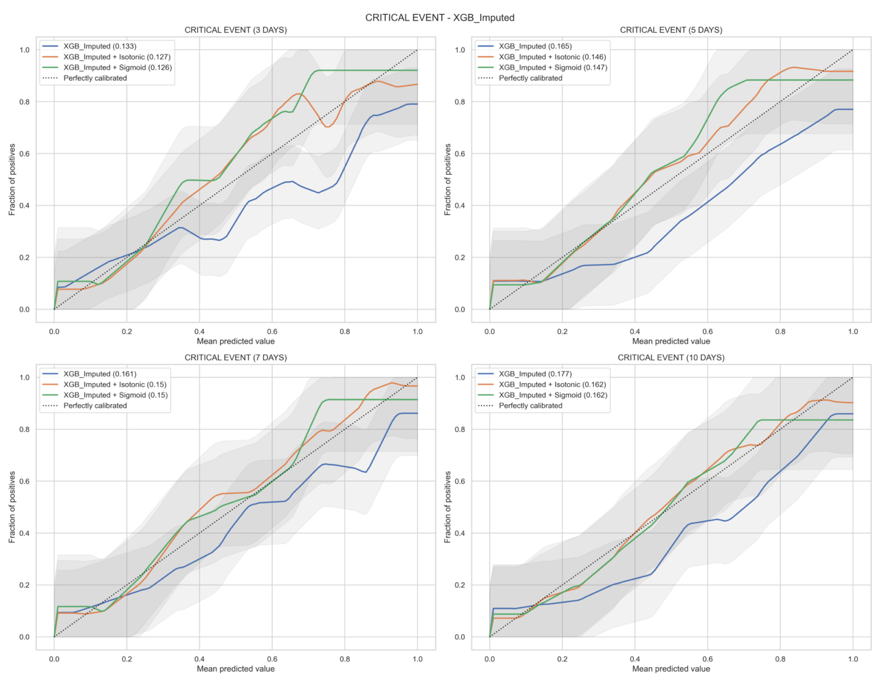


Figure S4: XGBoost imputed calibration plots predicting mortality at 3, 5, 7, and 10 days.


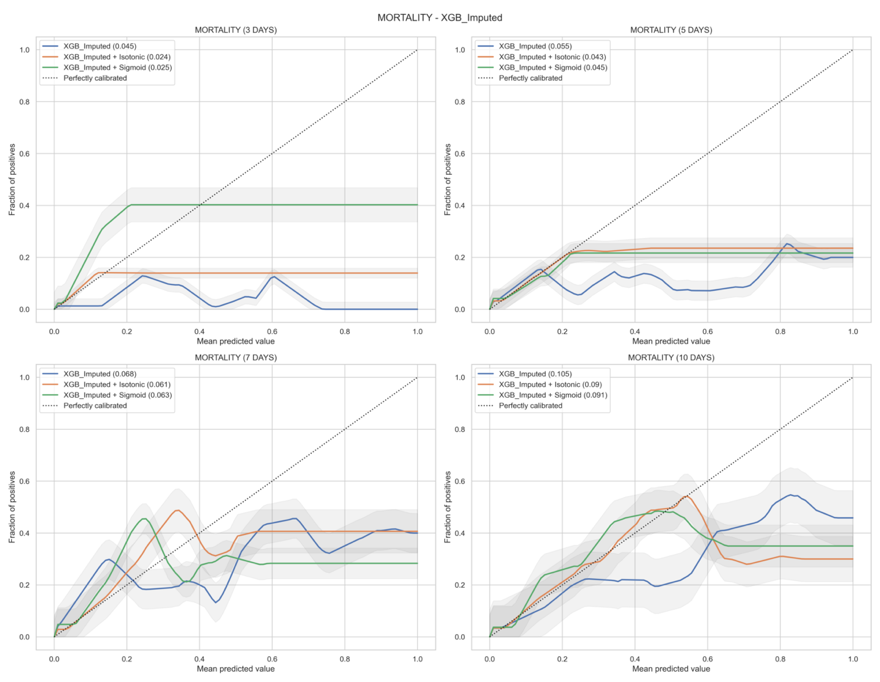


Figure S5: Logistic regression calibration plots predicting a critical event at 3, 5, 7, and 10 days.


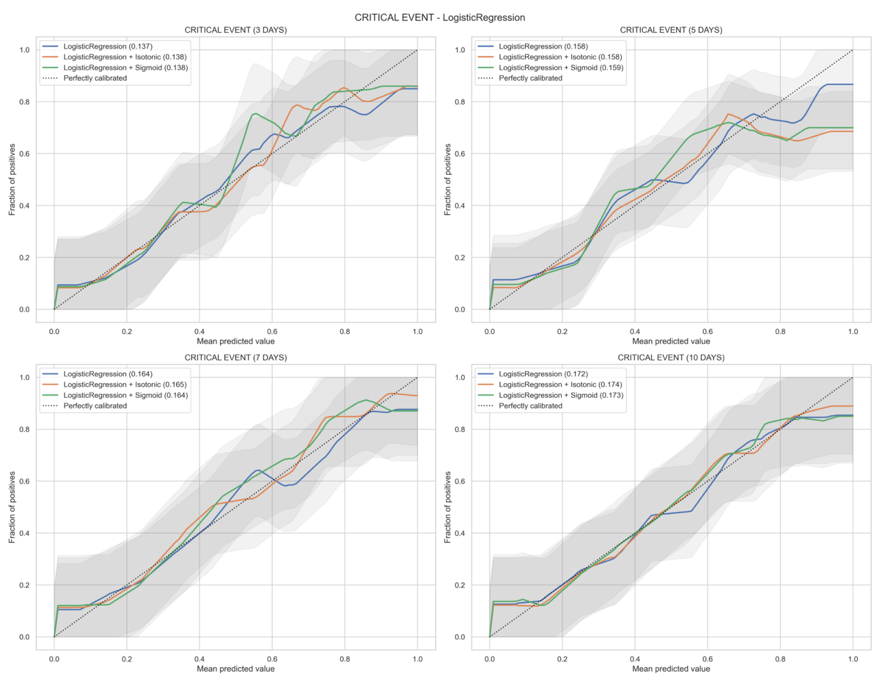


Figure S6: Logistic regression calibration plots predicting mortality at 3, 5, 7, and 10 days.


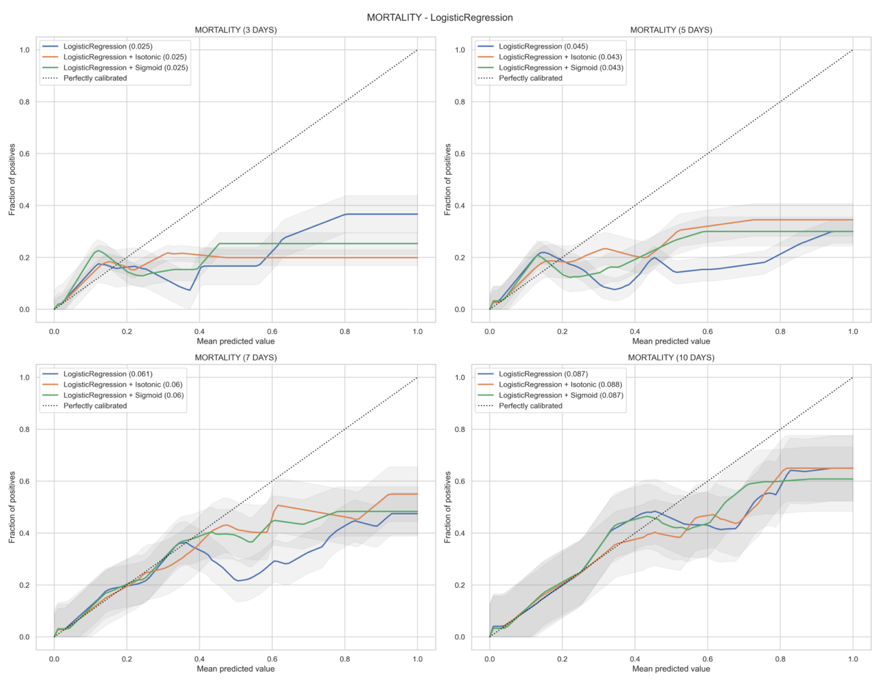


Figure S7: LASSO calibration plots predicting a critical event at 3, 5, 7, and 10 days.


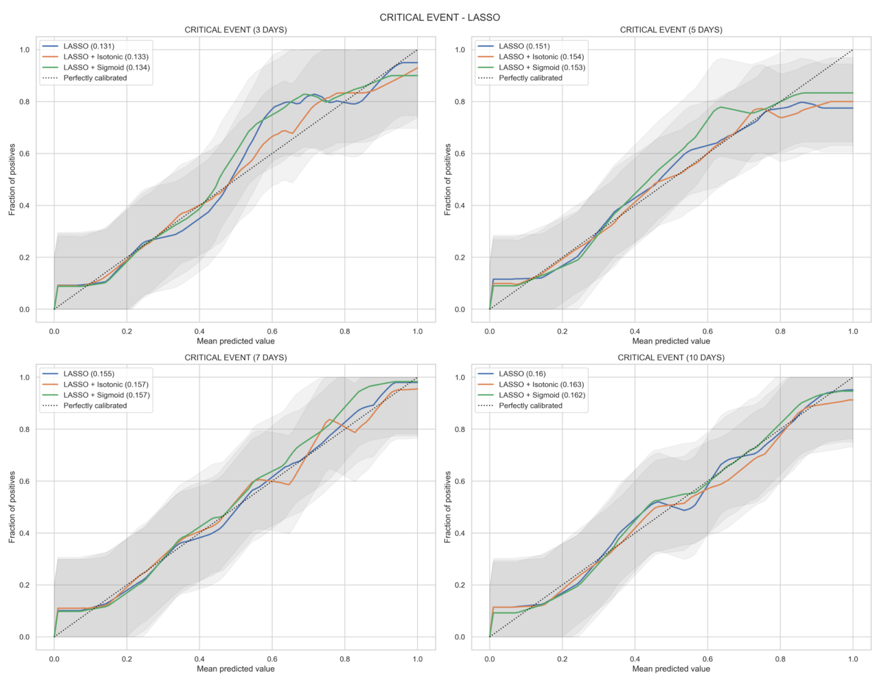


Figure S8: LASSO calibration plots predicting mortality at 3, 5, 7, and 10 days.


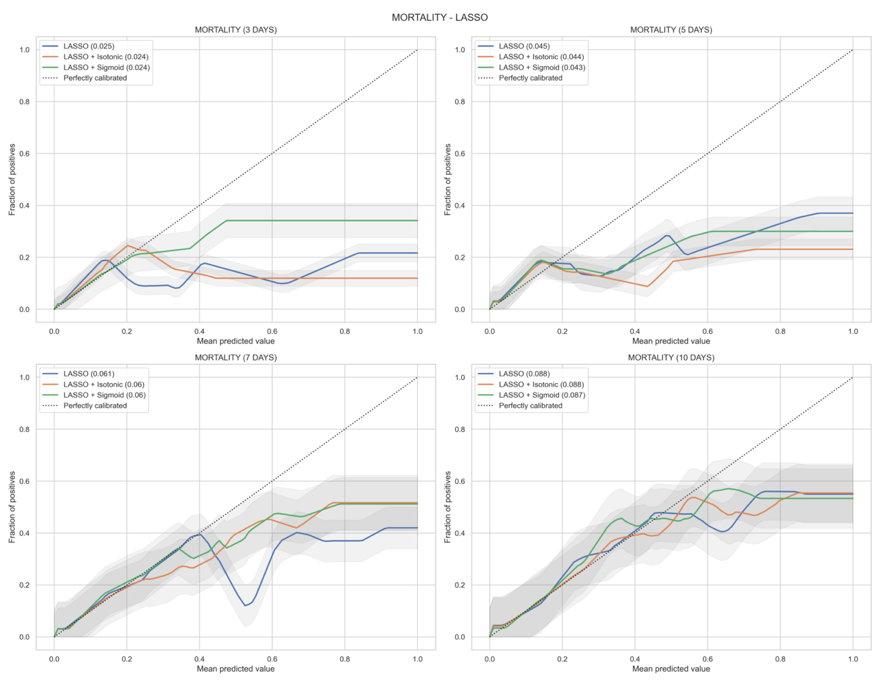


Figure S9: LASSO feature importance plot predicting critical event and mortality at day 7.


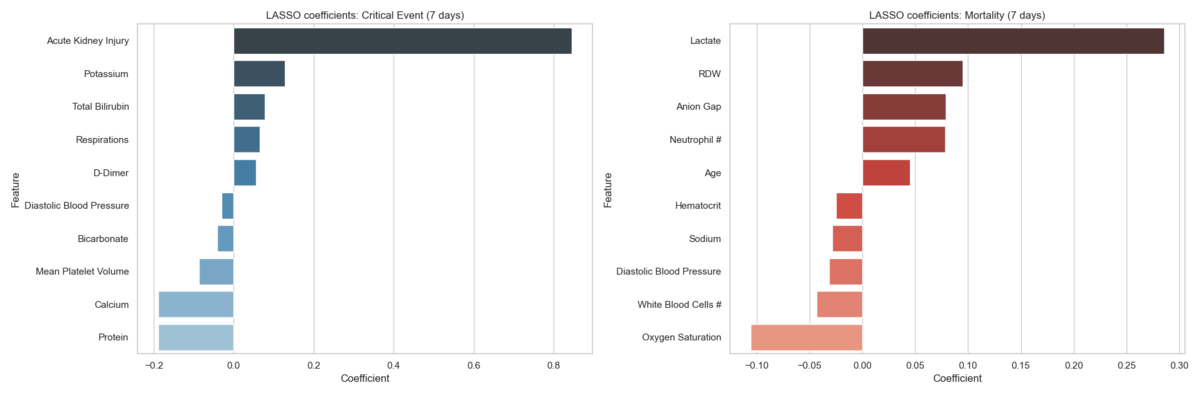


Figure S10: Survival curve for all patients with in-hospital mortality.


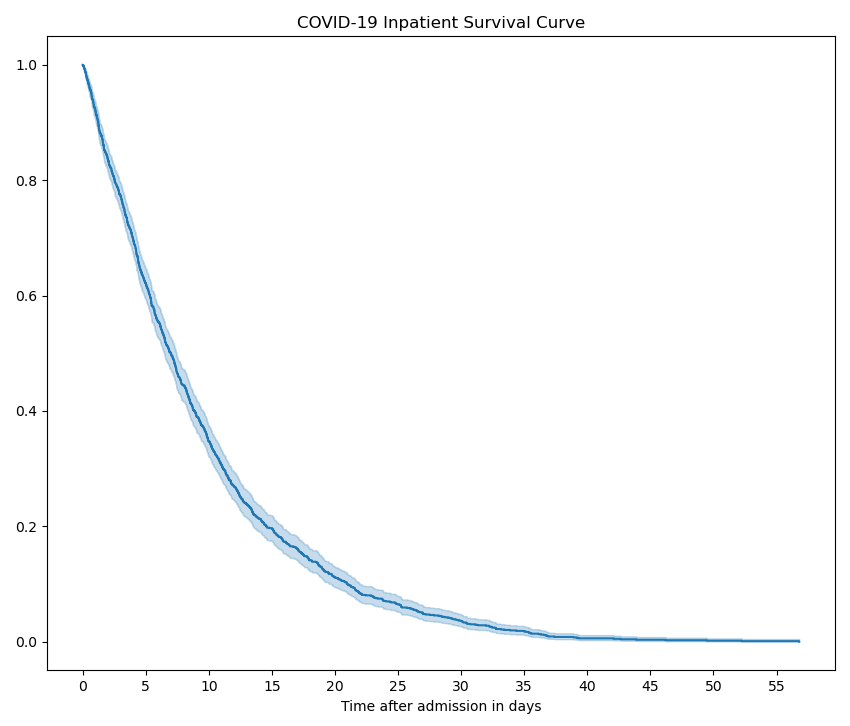


Figure S11: SHAP interaction scores predicting a critical event at 7 days.


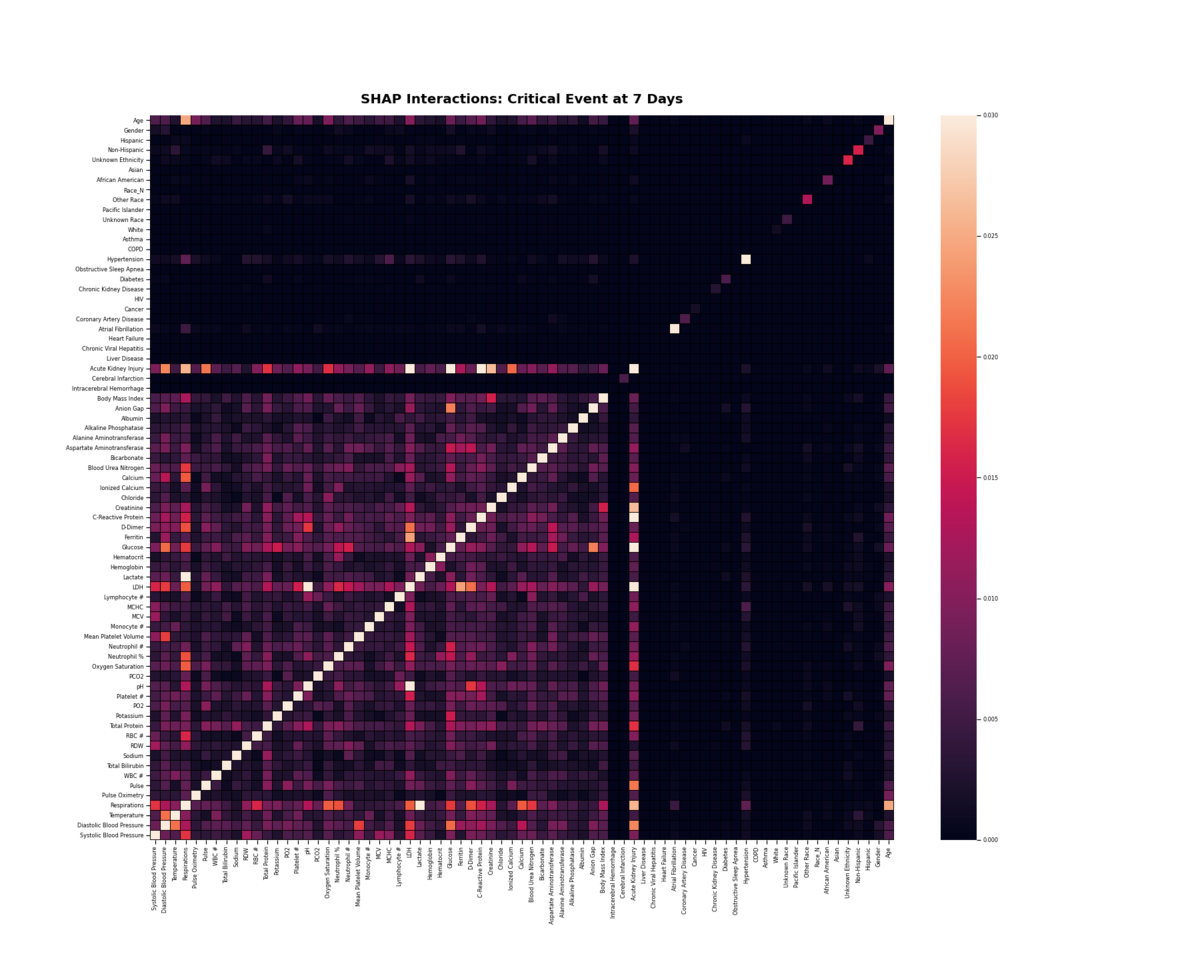


Figure S12: SHAP interaction scores predicting mortality at 7 days.


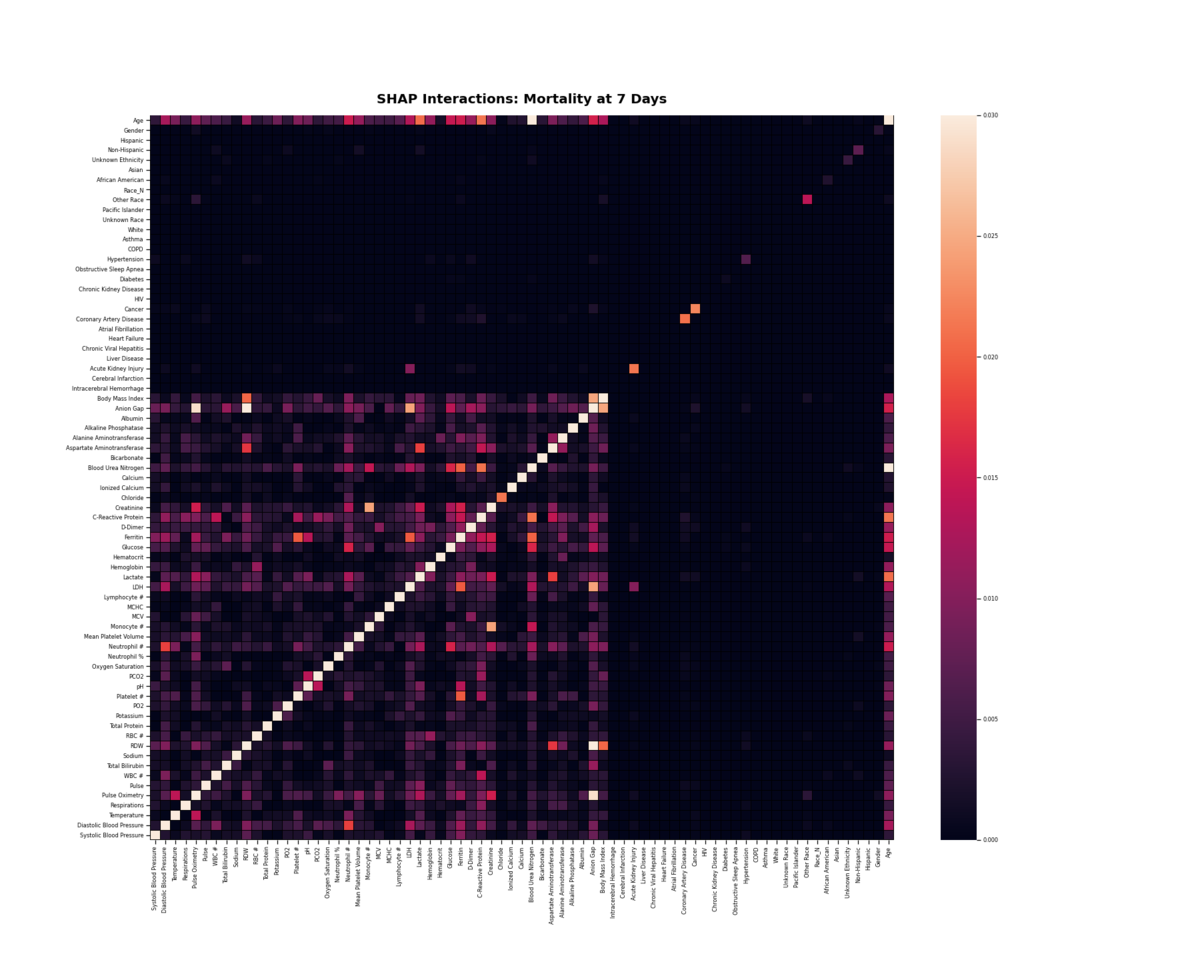

Supplement: Multimedia Appendix 2 [file jmir_v22i11e24018_app2.docx]
