# Supplementary material for: Machine Learning to Predict Mortality and Critical Events in a Cohort of Patients With COVID-19 in New York City: Model Development and Validation
Source: J Med Internet Res. 2020 Nov 6;22(11):e24018. doi: 10.2196/24018 (PMC7652593; doi:10.2196/24018)
Supplement: Multimedia Appendix 3 [file jmir_v22i11e24018_app3.docx]

**Supplementary Table 2: Brier Scores of Calibration Curves for each Model**

| **Model** | **Days** | **Outcome** | **Calibration Type** | **Brier score** |
| --- | --- | --- | --- | --- |
| **XGB_NotImputed** | 3 | Critical Event | XGB_NotImputed | 0.135 |
|  |  |  | XGB_NotImputed + Isotonic | 0.124 |
|  |  |  | XGB_NotImputed + Sigmoid | 0.124 |
|  | 5 |  | XGB_NotImputed | 0.156 |
|  |  |  | XGB_NotImputed + Isotonic | 0.143 |
|  |  |  | XGB_NotImputed + Sigmoid | 0.145 |
|  | 7 |  | XGB_NotImputed | 0.162 |
|  |  |  | XGB_NotImputed + Isotonic | 0.149 |
|  |  |  | XGB_NotImputed + Sigmoid | 0.149 |
|  | 10 |  | XGB_NotImputed | 0.171 |
|  |  |  | XGB_NotImputed + Isotonic | 0.161 |
|  |  |  | XGB_NotImputed + Sigmoid | 0.16 |
|  | 3 | Mortality | XGB_NotImputed | 0.022 |
|  |  |  | XGB_NotImputed + Isotonic | 0.019 |
|  |  |  | XGB_NotImputed + Sigmoid | 0.019 |
|  | 5 |  | XGB_NotImputed | 0.044 |
|  |  |  | XGB_NotImputed + Isotonic | 0.038 |
|  |  |  | XGB_NotImputed + Sigmoid | 0.039 |
|  | 7 |  | XGB_NotImputed | 0.061 |
|  |  |  | XGB_NotImputed + Isotonic | 0.054 |
|  |  |  | XGB_NotImputed + Sigmoid | 0.055 |
|  | 10 |  | XGB_NotImputed | 0.093 |
|  |  |  | XGB_NotImputed + Isotonic | 0.085 |
|  |  |  | XGB_NotImputed + Sigmoid | 0.085 |

| **XGB_Imputed** | 3 | Critical Event | XGB_Imputed | 0.133 |
| --- | --- | --- | --- | --- |
|  |  |  | XGB_Imputed + Isotonic | 0.127 |
|  |  |  | XGB_Imputed + Sigmoid | 0.126 |
|  | 5 |  | XGB_Imputed | 0.165 |
|  |  |  | XGB_Imputed + Isotonic | 0.146 |
|  |  |  | XGB_Imputed + Sigmoid | 0.147 |
|  | 7 |  | XGB_Imputed | 0.161 |
|  |  |  | XGB_Imputed + Isotonic | 0.15 |
|  |  |  | XGB_Imputed + Sigmoid | 0.15 |
|  | 10 |  | XGB_Imputed | 0.177 |
|  |  |  | XGB_Imputed + Isotonic | 0.162 |
|  |  |  | XGB_Imputed + Sigmoid | 0.162 |
|  | 3 | Mortality | XGB_Imputed | 0.045 |
|  |  |  | XGB_Imputed + Isotonic | 0.024 |
|  |  |  | XGB_Imputed + Sigmoid | 0.025 |
|  | 5 |  | XGB_Imputed | 0.055 |
|  |  |  | XGB_Imputed + Isotonic | 0.043 |
|  |  |  | XGB_Imputed + Sigmoid | 0.045 |
|  | 7 |  | XGB_Imputed | 0.068 |
|  |  |  | XGB_Imputed + Isotonic | 0.061 |
|  |  |  | XGB_Imputed + Sigmoid | 0.063 |
|  | 10 |  | XGB_Imputed | 0.105 |
|  |  |  | XGB_Imputed + Isotonic | 0.09 |
|  |  |  | XGB_Imputed + Sigmoid | 0.091 |

| **LogisticRegression** | 3 | Critical Event | LogisticRegression | 0.137 |
| --- | --- | --- | --- | --- |
|  |  |  | LogisticRegression + Isotonic | 0.138 |
|  |  |  | LogisticRegression + Sigmoid | 0.138 |
|  | 5 |  | LogisticRegression | 0.158 |
|  |  |  | LogisticRegression + Isotonic | 0.158 |
|  |  |  | LogisticRegression + Sigmoid | 0.159 |
|  | 7 |  | LogisticRegression | 0.164 |
|  |  |  | LogisticRegression + Isotonic | 0.165 |
|  |  |  | LogisticRegression + Sigmoid | 0.164 |
|  | 10 |  | LogisticRegression | 0.172 |
|  |  |  | LogisticRegression + Isotonic | 0.174 |
|  |  |  | LogisticRegression + Sigmoid | 0.173 |
|  | 3 | Mortality | LogisticRegression | 0.025 |
|  |  |  | LogisticRegression + Isotonic | 0.025 |
|  |  |  | LogisticRegression + Sigmoid | 0.025 |
|  | 5 |  | LogisticRegression | 0.045 |
|  |  |  | LogisticRegression + Isotonic | 0.043 |
|  |  |  | LogisticRegression + Sigmoid | 0.043 |
|  | 7 |  | LogisticRegression | 0.061 |
|  |  |  | LogisticRegression + Isotonic | 0.06 |
|  |  |  | LogisticRegression + Sigmoid | 0.06 |
|  | 10 |  | LogisticRegression | 0.087 |
|  |  |  | LogisticRegression + Isotonic | 0.88 |
|  |  |  | LogisticRegression + Sigmoid | 0.087 |

| **LASSO** | 3 | Critical Event | LASSO | 0.131 |
| --- | --- | --- | --- | --- |
|  |  |  | LASSO + Isotonic | 0.133 |
|  |  |  | LASSO + Sigmoid | 0.134 |
|  | 5 |  | LASSO | 0.151 |
|  |  |  | LASSO + Isotonic | 0.154 |
|  |  |  | LASSO + Sigmoid | 0.153 |
|  | 7 |  | LASSO | 0.155 |
|  |  |  | LASSO + Isotonic | 0.157 |
|  |  |  | LASSO + Sigmoid | 0.157 |
|  | 10 |  | LASSO | 0.16 |
|  |  |  | LASSO + Isotonic | 0.163 |
|  |  |  | LASSO + Sigmoid | 0.162 |
|  | 3 | Mortality | LASSO | 0.025 |
|  |  |  | LASSO + Isotonic | 0.024 |
|  |  |  | LASSO + Sigmoid | 0.024 |
|  | 5 |  | LASSO | 0.045 |
|  |  |  | LASSO + Isotonic | 0.044 |
|  |  |  | LASSO + Sigmoid | 0.043 |
|  | 7 |  | LASSO | 0.061 |
|  |  |  | LASSO + Isotonic | 0.06 |
|  |  |  | LASSO + Sigmoid | 0.06 |
|  | 10 |  | LASSO | 0.088 |
|  |  |  | LASSO + Isotonic | 0.088 |
|  |  |  | LASSO + Sigmoid | 0.087 |
