# Supplementary material for: Machine Learning to Predict Mortality and Critical Events in a Cohort of Patients With COVID-19 in New York City: Model Development and Validation
Source: J Med Internet Res. 2020 Nov 6;22(11):e24018. doi: 10.2196/24018 (PMC7652593; doi:10.2196/24018)
Supplement: Multimedia Appendix 4 [file jmir_v22i11e24018_app4.docx]

**Supplementary Table 3: TRIPOD Guidelines Report**

| Topic | # | Description | Manuscript Location |
| --- | --- | --- | --- |
| Title | 1 | Machine Learning to Predict Mortality and Critical Events in COVID-19 Positive New York City Patients   - Identifies the target population (COVID-19 Positive New York City Patients), outcome (Mortality and Critical Events) and the prediction model (Machine Learning) | Title |
| Abstract | 2 | Objective - We analyze Electronic Health Records from COVID-19 positive hospitalized patients admitted to the Mount Sinai Health System in New York City (NYC). We present machine learning models for making predictions about the hospital course over clinically meaningful time horizons based on patient characteristics at admission. We assess performance of these models at multiple hospitals and time points.  Study design- Utilized XGBoost and baseline comparator models. Models were trained on patients from MSH and then externally validated on patients on MSH and from four other hospitals before or on May 1, 2020 (retrospectively) and prospectively after May 1, 2020.  Setting- 5 hospitals in the Mount Sinai Health System  Participants- New York City confirmed COVID-19 positive patients  Sample size- 4098 patients  Predictors- demographics, comorbidities, lab test results, vital signs  Outcome- Predicting in-hospital mortality and critical events (mortality, transfer to ICU, discharge to hospice, and/or intubation) at 3, 5, 7 and 10 days  Statistical analysis- XGBoost, Logistic Regression, LASSO  Results- On the training set, the XGBoost classifier outperformed baseline models, with area-under the receiver-operating-curve (AUC-ROC) for mortality at 0.89 at 3 days, 0.85 at 5 days, and 7 days, and 0.84 at 10 days and cross-validated AUC-PRC of 0.45 at 3 days, 0.33 at 5 days , 0.44 at 7 days and 0.48 at 10 days. AUC-ROC for predicting a critical event was 0.80 at 3 days, 0.79 at 5 days, 0.80 at 7 days, and 0.81 at 10 days with AUC-PRC of 0.61 at 3 days, 0.62 at 5 days, 0.66 at 7 days and 0.70 at 10 days. The trends in external and prospective validation sets was also similar to that of the training set.  Conclusion- Model identified at-risk patients and uncovers relationships predicting outcomes | Abstract |
| Background, Objectives | 3a | Medical context- Over 23 million people have tested positive for SARS-CoV-2 worldwide. Patients with COVID-19 demonstrate varying symptomatology, making triaging difficult  Prognostication with machine learning is poised to accomplish this, but has been limited by small sample sizes, lack of generalization to diverse populations, disparities in feature missingness, and potential for bias.  Rationale for developing model-  Many predictive models have met with success but only consider one of either demographic, clinical symptoms, or lab values, rather than all of these factors conjointly. Our model encompasses all these factors in predicting disease trajectory.  Recent studies are limited in scope and lack certain components such as prospective validation or evaluation of multiple models. Thus, we developed a boosted decision tree-based machine learning model and validated (externally and prospectively) on a new set of patients from all five hospitals | Introduction |
| Background, Objectives | 3b | Objective- We analyze Electronic Health Records from COVID-19 positive hospitalized patients admitted to the Mount Sinai Health System in New York City (NYC). We present machine learning models for making predictions about the hospital course over clinically meaningful time horizons based on patient characteristics at admission. We assess performance of these models at multiple hospitals and time points.  Development of a boosted decision tree-based machine learning model trained on electronic health records which was first externally validated using the algorithm to four other hospital centers and then proactively validated on a new set of patients from all 5 hospitals | Abstract |
| Sources of Data | 4a | Cohort of all COVID-19+ primary hospitalizations at five hospitals from MSHS, Electronic Health Records, Aggregated by the Mount Sinai COVID Informatics Center. | Results |
| Sources of Data | 4b | March 15, 2020 to May 22, 2020 | Results |
| Participants | 5a | Hospitalized patients at 5 NYC hospitals in the Mount Sinai Health System | Results |
| Participants | 5b | Patients >18 years of age with a positive SARS-CoV2 RT-PCR test that was placed within 48 hours of admission and were intubated <48 hours after admission. | Figure 1 |
| Participants | 5c | Treatments include full gamut of hospital events, but notably include intubation and ICU admission. | Definition of Outcome |
| Outcome | 6a | Critical event (intubation, discharge to hospice, or death) or mortality (death) at 3, 5, 7, and 10 days. | Results |
| Outcome | 6b | All models were trained and evaluated using 10-fold stratified cross-validation of patients from our main hospital and tested on the other hospitals (combined four hospital sites) We also tested on both the main hospital and four other sites on a date range that occurred after initial model development. | Experimental Evaluation |
| Predictors | 7a | Predictors included available patient demographics, medical history, vitals at intake, and labs on admission (within 48 hours). These were developed based on input from a team of front-line clinicians into what was clinically relevant and comprehensive. | Table 1 |
| Predictors | 7b | We built machine learning models based on XGBoost (without imputation), XGBoost (with imputation), logistic regression (with imputation), and LASSO (with imputation) | Model Development, Selection and Experimentation |
| Sample Size | 8 | The sample size differed based on time window and site but in total we had data for 4,098 total patients. | Figure 1 |
| Missing Data | 9 | For all models, features were removed if missing in >30% of patients. In the primary XGBoost model, no further modifications were made as the model directs missing values through split points to minimize loss. We performed k-nearest neighbors (k=5) imputation logistic regression, LASSO, and another XGBoost model. | Model development, Selection and Experimentation |
| Statistical analysis methods | 10a | All predictors were used in the XGB model for prediction. | Model development, Selection and Experimentation |
| Statistical analysis methods | 10b | Models used in this study included an XGBoost (with and without imputation), logistic regression, and logistic regression with LASSO. | Model development, Selection and Experimentation |
| Statistical analysis methods | 10c | Models were first externally validated on other hospital patients from March 15,2020 to May 1, 2020 and then prospectively validated on the main hospital and other hospitals (independently) patients admitted from May 1,2020 to May 22,2020 | Study Design |
| Statistical analysis methods | 10d | Measures used to compare models included model accuracy, sensitivity, specificity, AUC-ROC,AUC-PRC, and F1-statistic. | Experimental Evaluation |
| Statistical analysis methods | 10e | No model recalibration was performed after training. |  |
| Risk groups | 11 | No risk groups were created. |  |
| Development vs. validation | 12 | We developed the model on the main hospital during the date range from March 15, 2020 to May 1, 2020 and externally validated it on other hospitals within and after this date range and prospectively (after the date range) on the main hospital. | Study Design |
| Participants | 13a | Flow of participants- Relevant patient events (intubation, discharge to hospice care, or death) were recorded and subsets were constructed at 3,5,7 and 10-day intervals after admission. The number of patients without the outcome differed based on experiment and based on time window. | Figure 1, Table 2, Results |
| Participants | 13b | Demographics, Medical history, Vital signs, Admission laboratory parameters  Please see Supplementary table 1 for missing data for all predictors and outcomes (critical event and mortality) | Table 1  Supplementary Table 1 |
| Model development | 14a | We show the number of patients involved and the proportion of events in each experiment by time window in Table 2. | Table 2 |
|  | 14b |  |  |
| Model specification | 15a | This does not apply to our model but see the GitHub repository for model specifications and Supplementary Table 2 for Hyperparameters. | <https://github.com/HPIMS/COVID-ML-Prediction>, Supplementary Table 2 |
|  | 15b | The prediction model cannot be used on other cohorts directly, per se, but we do release code in order to replicate how to build the model off identical data. | https://github.com/HPIMS/COVID-ML-Prediction |
| Model performance | 16 | Please refer to paragraph 2 of “Classifier training and performance” for performance measures and Table 2 for corresponding Confidence Intervals | Results, Figure 2, Table 2 |
| Limitations | 18 | Details on study limitations include missingness present in admission labs, temporal evolution of COVID management and resource constraints, inherent limitations using EHRs and interhospital system policies affecting care such as shuttling COVID-19 cases to certain hospitals.. | Discussion |
| Interpretation | 19a | Please see the manuscript for full discussion. | Interpretation, Results, Discussion |
|  | 19b | Encouraging results in terms of AUC-ROC and AUC-PRC on validation experiments that were reflective of cross-validation performance on original development. | Results |
| Implications | 20 | Model may have utility in identifying patients at short-term risk of a critical event and help optimize resource management at time of admission. | Discussion |
| Supplementary | 21 | Information about baseline patient characteristics, feature analysis, final model hyperparameters, model performance, missingness, SHAP interactions, calibration plots for all the classifiers and TRIPOD guidelines | Main Figures 1-4, Tables 1-2, Supplementary Tables 1-3, Supplementary Figures 1-11 |
| Funding | 22 | This work was Supported by U54 TR001433-05, National Center for Advancing Translational Sciences, National Institutes of Health. The funder had no role in the writing of the manuscript or the decision to submit it for publication. | Funding |
