# Supplementary material for: Machine Learning to Predict Mortality and Critical Events in a Cohort of Patients With COVID-19 in New York City: Model Development and Validation
Source: J Med Internet Res. 2020 Nov 6;22(11):e24018. doi: 10.2196/24018 (PMC7652593; doi:10.2196/24018)
Supplement: Multimedia Appendix 5 [file jmir_v22i11e24018_app5.docx]

### **Supplementary Table 4: Baseline Feature Variability Across All Patients**

| **Variable** | **Missing**  **(N)** | **Present**  **(%)** | **Mean** | **Standard Deviation** | **IQR - 0%** | **IQR - 25%** | **IQR - 50%** | **IQR - 75%** | **IQR - 100%** | **Histogram** |
| --- | --- | --- | --- | --- | --- | --- | --- | --- | --- | --- |
| Age | 0 | 100.0% | 65.3 | 16.7 | 18.2 | 55.2 | 66.9 | 77.7 | 103 | ▁▃▇▇▂ |
| Gender | 0 | 100.0% | 0.6 | 0.5 | 0 | 0 | 1 | 1 | 1 | ▆▁▁▁▇ |
| Ethnic_Group_Hispanic/Latino | 0 | 100.0% | 0.3 | 0.4 | 0 | 0 | 0 | 1 | 1 | ▇▁▁▁▃ |
| Ethnic_Group_Non-Hispanic Latino | 0 | 100.0% | 0.6 | 0.5 | 0 | 0 | 1 | 1 | 1 | ▆▁▁▁▇ |
| Ethnic_Group_Unknown | 0 | 100.0% | 0.1 | 0.3 | 0 | 0 | 0 | 0 | 1 | ▇▁▁▁▁ |
| Race_Asian | 0 | 100.0% | 0.0 | 0.2 | 0 | 0 | 0 | 0 | 1 | ▇▁▁▁▁ |
| Race_African American | 0 | 100.0% | 0.3 | 0.5 | 0 | 0 | 0 | 1 | 1 | ▇▁▁▁▃ |
| Race_Native | 0 | 100.0% | 0.0 | 0.0 | 0 | 0 | 0 | 0 | 1 | ▇▁▁▁▁ |
| Race_Other | 0 | 100.0% | 0.4 | 0.5 | 0 | 0 | 0 | 1 | 1 | ▇▁▁▁▅ |
| Race_Pacific Islander | 0 | 100.0% | 0.0 | 0.0 | 0 | 0 | 0 | 0 | 1 | ▇▁▁▁▁ |
| Race_Unknown | 0 | 100.0% | 0.0 | 0.2 | 0 | 0 | 0 | 0 | 1 | ▇▁▁▁▁ |
| Race_Caucasian | 0 | 100.0% | 0.2 | 0.4 | 0 | 0 | 0 | 0 | 1 | ▇▁▁▁▂ |
| ASTHMA | 0 | 100.0% | 0.1 | 0.2 | 0 | 0 | 0 | 0 | 1 | ▇▁▁▁▁ |
| CHRONIC OBSTRUCTIVE PULMONARY DISEASE | 0 | 100.0% | 0.0 | 0.2 | 0 | 0 | 0 | 0 | 1 | ▇▁▁▁▁ |
| HYPERTENSION | 0 | 100.0% | 0.3 | 0.5 | 0 | 0 | 0 | 1 | 1 | ▇▁▁▁▅ |
| OBSTRUCTIVE_SLEEP_APNEA | 0 | 100.0% | 0.0 | 0.1 | 0 | 0 | 0 | 0 | 1 | ▇▁▁▁▁ |
| DIABETES | 0 | 100.0% | 0.2 | 0.4 | 0 | 0 | 0 | 0 | 1 | ▇▁▁▁▂ |
| CHRONIC KIDNEY DISEASE | 0 | 100.0% | 0.1 | 0.3 | 0 | 0 | 0 | 0 | 1 | ▇▁▁▁▁ |
| HIV_FLAG | 0 | 100.0% | 0.0 | 0.1 | 0 | 0 | 0 | 0 | 1 | ▇▁▁▁▁ |
| CANCER | 0 | 100.0% | 0.1 | 0.3 | 0 | 0 | 0 | 0 | 1 | ▇▁▁▁▁ |
| CORONARY ARTERY DISEASE | 0 | 100.0% | 0.1 | 0.3 | 0 | 0 | 0 | 0 | 1 | ▇▁▁▁▁ |
| ATRIAL FIBRILLATION | 0 | 100.0% | 0.1 | 0.3 | 0 | 0 | 0 | 0 | 1 | ▇▁▁▁▁ |
| HEART FAILURE | 0 | 100.0% | 0.1 | 0.3 | 0 | 0 | 0 | 0 | 1 | ▇▁▁▁▁ |
| CHRONIC_VIRAL_HEPATITIS | 0 | 100.0% | 0.0 | 0.1 | 0 | 0 | 0 | 0 | 1 | ▇▁▁▁▁ |
| ALCOHOLIC_NONALCOHOLIC_LIVER_DISEASE | 0 | 100.0% | 0.0 | 0.2 | 0 | 0 | 0 | 0 | 1 | ▇▁▁▁▁ |
| ACUTE_KIDNEY_INJURY | 0 | 100.0% | 0.2 | 0.4 | 0 | 0 | 0 | 0 | 1 | ▇▁▁▁▂ |
| CEREBRAL_INFARCTION | 0 | 100.0% | 0.0 | 0.2 | 0 | 0 | 0 | 0 | 1 | ▇▁▁▁▁ |
| INTRACEREBRAL_HEMORRHAGE | 0 | 100.0% | 0.0 | 0.1 | 0 | 0 | 0 | 0 | 1 | ▇▁▁▁▁ |
| BMI | 285 | 93.0% | 29.2 | 9.3 | 1.63 | 24.2 | 27.7 | 32.6 | 218 | ▇▁▁▁▁ |
| AGAP | 94 | 97.7% | 12.3 | 3.3 | 4.4 | 10 | 12 | 14 | 27.4 | ▂▇▃▁▁ |
| ALBUMIN | 296 | 92.8% | 2.9 | 0.5 | 1.1 | 2.5 | 2.9 | 3.2 | 4.1 | ▁▃▆▇▃ |
| ALKPHOS | 297 | 92.8% | 93.3 | 68.0 | 35 | 59 | 76 | 102 | 967 | ▇▁▁▁▁ |
| ALANINE AMINOTRANSFERASE | 298 | 92.7% | 50.0 | 76.7 | 7 | 18 | 30 | 53 | 1163 | ▇▁▁▁▁ |
| AMYLASE | 4008 | 2.2% | 111.0 | 105.0 | 21 | 52 | 81.5 | 122 | 620 | ▇▂▁▁▁ |
| ASPARTATE AMINOTRANSFERASE | 304 | 92.6% | 67.4 | 104.0 | 11 | 28 | 42 | 70 | 1413 | ▇▁▁▁▁ |
| ATYPLYMPH | 3452 | 15.8% | 3.9 | 5.1 | 1 | 1 | 2 | 4 | 36 | ▇▁▁▁▁ |
| BANDS | 3019 | 26.3% | 7.9 | 6.8 | 2 | 3 | 6 | 10 | 42 | ▇▂▁▁▁ |
| BASOPHIL | 2709 | 33.9% | 0.1 | 0.0 | 0.1 | 0.1 | 0.1 | 0.1 | 0.1 | ▁▁▇▁▁ |
| BASOPHIL_PERC | 2403 | 41.4% | 0.4 | 0.3 | 0.1 | 0.1 | 0.3 | 0.5 | 1.9 | ▇▂▁▁▁ |
| BICARB | 87 | 97.9% | 23.2 | 4.3 | 11.4 | 20.9 | 23 | 26 | 41.2 | ▂▇▇▁▁ |
| BICARB_A | 3053 | 25.5% | 23.7 | 5.2 | 11.3 | 20 | 23.9 | 27 | 43.8 | ▂▇▇▁▁ |
| BLASTS | 4075 | 0.6% | 8.7 | 18.9 | 1 | 1 | 1 | 4 | 84 | ▇▁▁▁▁ |
| BNP | 3551 | 13.3% | 487.0 | 1169.0 | 10.4 | 44 | 110 | 403 | 11970 | ▇▁▁▁▁ |
| BUN | 127 | 96.9% | 29.0 | 25.8 | 6 | 12 | 19 | 36 | 174 | ▇▂▁▁▁ |
| CALCIUM | 99 | 97.6% | 8.2 | 0.7 | 5.9 | 7.8 | 8.2 | 8.6 | 10.5 | ▁▂▇▃▁ |
| CALCIUMIONIZED | 2544 | 37.9% | 1.2 | 0.1 | 0.85 | 1.11 | 1.16 | 1.21 | 1.51 | ▁▃▇▂▁ |
| CALCIUMIONIZED_A | 3386 | 17.4% | 1.2 | 0.1 | 0.82 | 1.09 | 1.15 | 1.21 | 1.52 | ▁▃▇▂▁ |
| CHLORIDE | 96 | 97.7% | 104.0 | 6.3 | 87 | 100 | 104 | 107 | 128 | ▁▇▇▂▁ |
| CHLORIDE_A | 3640 | 11.2% | 104.0 | 6.3 | 92 | 99 | 103 | 108 | 124 | ▃▇▇▂▁ |
| CKMB | 3829 | 6.6% | 9.2 | 22.3 | 0.3 | 1.7 | 3.4 | 8.1 | 260 | ▇▁▁▁▁ |
| CREATININE PHOSPHOKINASE | 3325 | 18.9% | 566.0 | 1489.0 | 13 | 71 | 160 | 477 | 15919 | ▇▁▁▁▁ |
| CREATININE | 87 | 97.9% | 1.8 | 2.2 | 0.3 | 0.74 | 0.97 | 1.61 | 14.9 | ▇▁▁▁▁ |
| C- REACTIVE PROTEIN | 894 | 78.2% | 139.0 | 98.0 | 0.78 | 58.1 | 120 | 208 | 455 | ▇▆▅▂▁ |
| DBILIRUBIN | 2916 | 28.8% | 0.5 | 0.9 | 0.2 | 0.2 | 0.3 | 0.5 | 11 | ▇▁▁▁▁ |
| D-DIMER | 1857 | 54.7% | 3.2 | 3.8 | 0.29 | 0.89 | 1.69 | 3.51 | 19 | ▇▁▁▁▁ |
| EOSINO | 1515 | 63.0% | 0.1 | 0.1 | 0.1 | 0.1 | 0.1 | 0.1 | 1 | ▇▁▁▁▁ |
| EOSINO_PERC | 2373 | 42.1% | 0.7 | 1.1 | 0.1 | 0.1 | 0.3 | 0.8 | 11.2 | ▇▁▁▁▁ |
| FERRITIN | 1712 | 58.2% | 1546.0 | 2207.0 | 22 | 371 | 814 | 1916 | 19186 | ▇▁▁▁▁ |
| FIBRINOGEN | 2824 | 31.1% | 617.0 | 197.0 | 101 | 477 | 612 | 746 | 1195 | ▁▆▇▃▁ |
| GLUCOSE | 88 | 97.9% | 139.0 | 74.2 | 51 | 94 | 112 | 157 | 534 | ▇▂▁▁▁ |
| GLUCOSE_A | 3395 | 17.2% | 186.0 | 81.3 | 61 | 125 | 163 | 225 | 449 | ▇▇▃▂▁ |
| HCT | 92 | 97.8% | 37.6 | 6.6 | 17.7 | 33.4 | 38.1 | 42.3 | 52.8 | ▁▃▇▇▂ |
| HCT_A | 3668 | 10.5% | 36.4 | 7.2 | 20 | 31 | 37 | 41 | 55 | ▃▅▇▅▁ |
| HGB | 102 | 97.5% | 12.1 | 2.1 | 5.9 | 10.8 | 12.4 | 13.7 | 16.7 | ▁▃▆▇▂ |
| HGB_A | 3656 | 10.8% | 12.2 | 2.4 | 6.5 | 10.4 | 12.4 | 13.9 | 18.4 | ▂▅▇▅▁ |
| IL6 | 3609 | 11.9% | 165.0 | 330.0 | 1.8 | 30.9 | 67.9 | 155 | 2642 | ▇▁▁▁▁ |
| INR | 2179 | 46.8% | 1.3 | 0.5 | 1 | 1.1 | 1.2 | 1.3 | 5.1 | ▇▁▁▁▁ |
| IRON | 3730 | 9.0% | 42.9 | 33.3 | 8 | 20 | 32.5 | 54.2 | 200 | ▇▂▁▁▁ |
| LACTATE | 1733 | 57.7% | 1.9 | 1.0 | 0.7 | 1.2 | 1.6 | 2.2 | 10.1 | ▇▁▁▁▁ |
| LACTATE_A | 3386 | 17.4% | 1.8 | 1.4 | 0.5 | 1 | 1.4 | 2.1 | 10.1 | ▇▁▁▁▁ |
| LACTATE DEHYDROGENASE | 1838 | 55.1% | 493.0 | 262.0 | 155 | 320 | 433 | 587 | 2482 | ▇▂▁▁▁ |
| LYMPHO | 364 | 91.1% | 1.1 | 0.6 | 0.2 | 0.7 | 1 | 1.4 | 5.3 | ▇▃▁▁▁ |
| LYMPHO_PERC | 2355 | 42.5% | 16.1 | 9.5 | 1.5 | 8.7 | 14.2 | 21.4 | 52.9 | ▇▇▃▁▁ |
| MCHC | 100 | 97.6% | 29.5 | 2.4 | 21.6 | 28.1 | 29.7 | 31.1 | 35.9 | ▁▃▇▇▁ |
| MCV | 118 | 97.1% | 90.4 | 6.9 | 66.5 | 86.5 | 90.8 | 94.6 | 110 | ▁▂▇▆▁ |
| MONO | 361 | 91.2% | 0.5 | 0.3 | 0.1 | 0.3 | 0.5 | 0.7 | 2 | ▇▇▂▁▁ |
| MONO_PERC | 2354 | 42.6% | 7.1 | 3.6 | 0.9 | 4.3 | 6.5 | 9.2 | 19.9 | ▆▇▅▁▁ |
| MPV | 121 | 97.0% | 8.7 | 1.3 | 6.2 | 7.8 | 8.5 | 9.4 | 13.4 | ▃▇▅▁▁ |
| NEUTRO | 363 | 91.1% | 6.9 | 4.3 | 1.1 | 3.7 | 5.7 | 8.8 | 28.7 | ▇▃▁▁▁ |
| NEUTRO_PERC | 269 | 93.4% | 75.8 | 12.8 | 25.3 | 68.1 | 78.2 | 85.5 | 96.9 | ▁▁▃▇▇ |
| O2SAT | 2523 | 38.4% | 75.3 | 21.6 | 15.9 | 62.4 | 81.3 | 93.3 | 99.7 | ▁▂▂▃▇ |
| O2SAT_A | 3235 | 21.1% | 95.3 | 5.8 | 63.7 | 94 | 97.5 | 99 | 100 | ▁▁▁▂▇ |
| PCO2 | 2505 | 38.9% | 43.4 | 10.1 | 23 | 37 | 42 | 48 | 94.2 | ▃▇▂▁▁ |
| PCO2_A | 3201 | 21.9% | 43.9 | 15.0 | 16.5 | 34 | 40 | 51 | 100 | ▃▇▃▁▁ |
| PH | 2508 | 38.8% | 7.4 | 0.1 | 7.03 | 7.35 | 7.39 | 7.43 | 7.54 | ▁▁▂▇▃ |
| PH_A | 3047 | 25.6% | 7.4 | 0.1 | 6.95 | 7.27 | 7.37 | 7.44 | 7.55 | ▁▂▅▇▆ |
| PLT | 105 | 97.4% | 232.0 | 105.0 | 25 | 161 | 214 | 286 | 725 | ▅▇▂▁▁ |
| PO2 | 2558 | 37.6% | 57.5 | 33.6 | 18 | 36 | 49 | 67 | 200 | ▇▃▁▁▁ |
| PO2_A | 3054 | 25.5% | 114.0 | 68.4 | 36.8 | 67 | 90 | 136 | 394 | ▇▃▁▁▁ |
| POTASSIUM | 94 | 97.7% | 4.2 | 0.7 | 2.8 | 3.8 | 4.2 | 4.6 | 6.9 | ▃▇▅▁▁ |
| POTASSIUM_A | 3387 | 17.3% | 4.3 | 0.8 | 2.4 | 3.7 | 4.2 | 4.85 | 6.8 | ▂▇▇▃▁ |
| PROTEIN | 297 | 92.8% | 6.4 | 0.7 | 3.8 | 6 | 6.4 | 6.9 | 8.8 | ▁▃▇▃▁ |
| PROTHROMBIN TIME | 2164 | 47.2% | 15.8 | 4.0 | 12.3 | 13.7 | 14.7 | 16.3 | 45.9 | ▇▁▁▁▁ |
| PARTIAL PROTHROMBIN TIME | 2190 | 46.6% | 38.4 | 17.5 | 21.9 | 29.7 | 33.6 | 39.7 | 155 | ▇▁▁▁▁ |
| RBC CNT | 105 | 97.4% | 4.1 | 0.7 | 1.9 | 3.7 | 4.22 | 4.66 | 5.97 | ▁▃▇▇▂ |
| RED BLOOD CELL DISTRIBUTION WIDTH | 139 | 96.6% | 14.3 | 2.4 | 11.2 | 12.6 | 13.7 | 15.1 | 26.8 | ▇▃▁▁▁ |
| SODIUM | 89 | 97.8% | 139.0 | 5.8 | 124 | 136 | 138 | 141 | 162 | ▁▇▅▁▁ |
| SODIUM_A | 3386 | 17.4% | 137.0 | 6.2 | 125 | 133 | 136 | 141 | 156 | ▅▇▆▂▁ |
| TOTAL BILIRUBIN | 364 | 91.1% | 0.7 | 0.6 | 0.3 | 0.4 | 0.6 | 0.8 | 11.1 | ▇▁▁▁▁ |
| TIBC | 3735 | 8.9% | 180.0 | 55.4 | 68 | 140 | 172 | 210 | 426 | ▃▇▃▁▁ |
| TRANSFERRINSAT | 3867 | 5.6% | 22.4 | 14.6 | 4 | 11 | 19 | 28 | 75 | ▇▆▂▂▁ |
| TROPONIN I | 2227 | 45.7% | 0.5 | 1.9 | 0.01 | 0.02 | 0.059 | 0.184 | 23.4 | ▇▁▁▁▁ |
| URICACID | 4084 | 0.3% | 6.0 | 0.9 | 4 | 5.45 | 5.95 | 6.42 | 7.6 | ▂▅▇▃▅ |
| WBC | 101 | 97.5% | 8.7 | 5.1 | 1.3 | 5.3 | 7.4 | 10.7 | 50.2 | ▇▂▁▁▁ |
| CAC..PULSE (HEART RATE) | 1 | 100.0% | 87.2 | 16.2 | 47 | 76 | 86 | 97 | 150 | ▂▇▆▂▁ |
| CAC..PULSE.OXIMETRY | 13 | 99.7% | 95.6 | 2.9 | 72 | 94 | 96 | 98 | 99 | ▁▁▁▂▇ |
| CAC..RESPIRATORY RATE | 1 | 100.0% | 20.1 | 4.2 | 6 | 18 | 19 | 20 | 46 | ▁▇▁▁▁ |
| CAC..TEMPERATURE | 1 | 100.0% | 98.7 | 1.4 | 95.1 | 97.8 | 98.5 | 99.5 | 103 | ▁▇▇▂▁ |
| DIASTOLIC BLOOD PRESSURE | 2 | 100.0% | 71.3 | 12.0 | 38 | 63 | 71 | 79 | 105 | ▁▅▇▅▁ |
| SYSTOLIC BLOOD PRESSURE | 2 | 100.0% | 127.0 | 20.8 | 71 | 112 | 125 | 140 | 189 | ▁▇▇▃▁ |
