# Supplementary material for: Machine Learning to Predict Mortality and Critical Events in a Cohort of Patients With COVID-19 in New York City: Model Development and Validation
Source: J Med Internet Res. 2020 Nov 6;22(11):e24018. doi: 10.2196/24018 (PMC7652593; doi:10.2196/24018)
Supplement: Multimedia Appendix 6 [file jmir_v22i11e24018_app6.docx]

**Supplementary Table 5: Model Performance by Experiment.**

Performance of the XGBoost classifier by hospital site, as measured by accuracy (ACC), area under the receiver operating curve (AUC-ROC), area under the precision recall curve (AU-PRC), F1-score (F1), sensitivity (SENS), and specificity (SPEC). “OH” refers to all hospitals in the external validation set (ie, MSW, MSM, MSB, MSQ). Outcomes are structured by “<outcome>_<day>”, where outcome is either a critical event (CRITICAL) or mortality (MORTALITY) and the time frame it was predicted over. “OUTCOME PERC” refers to the proportion of the data set with the respective outcome. We used two time points for these experiments based on an initial enrollment freeze. All endpoints were allowed to occur for patients admitted before the enrollment freeze. For the MSH experiment in the first time point, there were seven patients in which we were unable to obtain ultimate outcomes due to data migration issues.

| **EXPERIMENT** | **OUTCOME** | **N** | **CLASSIFIER** | **POSITIVE OUTCOMES** | **OUTCOME PROPORTION** | **ACCURACY** | **AUC-ROC** | **AU-PRC** | **F1S** | **SENS** | **SPEC** |
| --- | --- | --- | --- | --- | --- | --- | --- | --- | --- | --- | --- |
| **MSH > MSH** | **CRITICAL_3** | **1514** | LASSO | 322 | 0.213 | 0.779 (0.775 - 0.783) | 0.751 (0.749 - 0.754) | 0.536 (0.532 - 0.54) | 0.52 (0.517 - 0.523) | 0.558 (0.551 - 0.566) | 0.839 (0.833 - 0.845) |
|  |  |  | LogisticRegression | 322 | 0.213 | 0.778 (0.775 - 0.781) | 0.752 (0.749 - 0.754) | 0.536 (0.533 - 0.54) | 0.52 (0.517 - 0.523) | 0.561 (0.554 - 0.568) | 0.837 (0.831 - 0.843) |
|  |  |  | XGB_Imputed | 322 | 0.213 | 0.807 (0.805 - 0.81) | 0.788 (0.786 - 0.79) | 0.586 (0.583 - 0.59) | 0.573 (0.57 - 0.576) | 0.605 (0.599 - 0.611) | 0.862 (0.858 - 0.866) |
|  |  |  | **XGB_Unimputed** | 322 | 0.213 | 0.816 (0.813 - 0.818) | 0.795 (0.793 - 0.797) | 0.604 (0.601 - 0.608) | 0.583 (0.58 - 0.585) | 0.602 (0.596 - 0.608) | 0.874 (0.869 - 0.878) |
|  | **CRITICAL_5** | **1512** | LASSO | 386 | 0.255 | 0.736 (0.732 - 0.739) | 0.747 (0.745 - 0.749) | 0.555 (0.551 - 0.558) | 0.55 (0.547 - 0.552) | 0.629 (0.623 - 0.635) | 0.772 (0.766 - 0.778) |
|  |  |  | LogisticRegression | 386 | 0.255 | 0.735 (0.732 - 0.738) | 0.747 (0.745 - 0.749) | 0.555 (0.551 - 0.558) | 0.55 (0.548 - 0.552) | 0.632 (0.626 - 0.638) | 0.77 (0.764 - 0.776) |
|  |  |  | XGB_Imputed | 386 | 0.255 | 0.763 (0.761 - 0.766) | 0.78 (0.778 - 0.782) | 0.598 (0.595 - 0.601) | 0.589 (0.587 - 0.592) | 0.664 (0.658 - 0.67) | 0.798 (0.793 - 0.803) |
|  |  |  | **XGB_Unimputed** | 386 | 0.255 | 0.773 (0.771 - 0.776) | 0.788 (0.786 - 0.789) | 0.618 (0.615 - 0.621) | 0.597 (0.594 - 0.599) | 0.655 (0.649 - 0.66) | 0.814 (0.809 - 0.819) |
|  | **CRITICAL_7** | **1510** | LASSO | 437 | 0.289 | 0.734 (0.731 - 0.737) | 0.765 (0.763 - 0.767) | 0.611 (0.608 - 0.614) | 0.592 (0.59 - 0.594) | 0.665 (0.659 - 0.67) | 0.763 (0.757 - 0.769) |
|  |  |  | LogisticRegression | 437 | 0.289 | 0.734 (0.731 - 0.737) | 0.765 (0.763 - 0.767) | 0.611 (0.608 - 0.614) | 0.592 (0.59 - 0.594) | 0.665 (0.659 - 0.67) | 0.763 (0.757 - 0.769) |
|  |  |  | XGB_Imputed | 437 | 0.289 | 0.763 (0.76 - 0.765) | 0.802 (0.8 - 0.804) | 0.659 (0.656 - 0.662) | 0.634 (0.632 - 0.636) | 0.71 (0.704 - 0.715) | 0.784 (0.779 - 0.789) |
|  |  |  | **XGB_Unimputed** | 437 | 0.289 | 0.765 (0.762 - 0.767) | 0.804 (0.802 - 0.806) | 0.664 (0.661 - 0.666) | 0.634 (0.632 - 0.636) | 0.705 (0.699 - 0.71) | 0.789 (0.784 - 0.794) |
|  | **CRITICAL_10** | **1507** | LASSO | 496 | 0.329 | 0.723 (0.72 - 0.725) | 0.77 (0.768 - 0.772) | 0.65 (0.647 - 0.652) | 0.625 (0.623 - 0.627) | 0.701 (0.696 - 0.706) | 0.733 (0.728 - 0.739) |
|  |  |  | LogisticRegression | 496 | 0.329 | 0.724 (0.722 - 0.727) | 0.77 (0.768 - 0.772) | 0.649 (0.646 - 0.652) | 0.625 (0.623 - 0.627) | 0.698 (0.693 - 0.703) | 0.737 (0.731 - 0.742) |
|  |  |  | XGB_Imputed | 496 | 0.329 | 0.757 (0.755 - 0.759) | 0.802 (0.8 - 0.804) | 0.692 (0.69 - 0.695) | 0.663 (0.661 - 0.665) | 0.726 (0.721 - 0.731) | 0.772 (0.767 - 0.777) |
|  |  |  | **XGB_Unimputed** | 496 | 0.329 | 0.759 (0.757 - 0.762) | 0.808 (0.807 - 0.81) | 0.7 (0.698 - 0.703) | 0.667 (0.666 - 0.669) | 0.733 (0.728 - 0.738) | 0.773 (0.768 - 0.777) |
|  | **MORTALITY_3** | **1514** | LASSO | 40 | 0.026 | 0.944 (0.942 - 0.947) | 0.797 (0.791 - 0.802) | 0.136 (0.13 - 0.142) | 0.24 (0.233 - 0.247) | 0.308 (0.296 - 0.32) | 0.962 (0.959 - 0.965) |
|  |  |  | LogisticRegression | 40 | 0.026 | 0.944 (0.941 - 0.947) | 0.805 (0.8 - 0.811) | 0.142 (0.136 - 0.148) | 0.241 (0.234 - 0.248) | 0.311 (0.298 - 0.324) | 0.961 (0.958 - 0.964) |
|  |  |  | XGB_Imputed | 40 | 0.026 | 0.915 (0.909 - 0.92) | 0.806 (0.801 - 0.81) | 0.119 (0.114 - 0.124) | 0.213 (0.207 - 0.219) | 0.375 (0.36 - 0.391) | 0.93 (0.924 - 0.935) |
|  |  |  | **XGB_Unimputed** | 40 | 0.026 | 0.976 (0.976 - 0.977) | 0.89 (0.886 - 0.894) | 0.445 (0.435 - 0.454) | 0.498 (0.489 - 0.506) | 0.442 (0.432 - 0.453) | 0.991 (0.99 - 0.992) |
|  | **MORTALITY_5** | **1512** | LASSO | 74 | 0.049 | 0.9 (0.896 - 0.905) | 0.785 (0.781 - 0.789) | 0.19 (0.185 - 0.195) | 0.281 (0.276 - 0.286) | 0.384 (0.372 - 0.396) | 0.927 (0.922 - 0.931) |
|  |  |  | LogisticRegression | 74 | 0.049 | 0.898 (0.894 - 0.902) | 0.785 (0.782 - 0.789) | 0.191 (0.186 - 0.196) | 0.281 (0.276 - 0.286) | 0.393 (0.381 - 0.405) | 0.924 (0.919 - 0.928) |
|  |  |  | XGB_Imputed | 74 | 0.049 | 0.886 (0.882 - 0.89) | 0.821 (0.818 - 0.824) | 0.176 (0.172 - 0.18) | 0.293 (0.288 - 0.297) | 0.472 (0.46 - 0.484) | 0.907 (0.903 - 0.911) |
|  |  |  | **XGB_Unimputed** | 74 | 0.049 | 0.93 (0.928 - 0.932) | 0.847 (0.844 - 0.85) | 0.329 (0.322 - 0.336) | 0.376 (0.37 - 0.382) | 0.422 (0.411 - 0.433) | 0.956 (0.953 - 0.959) |
|  | **MORTALITY_7** | **1510** | LASSO | 112 | 0.074 | 0.886 (0.882 - 0.889) | 0.821 (0.818 - 0.823) | 0.316 (0.31 - 0.321) | 0.384 (0.379 - 0.389) | 0.462 (0.454 - 0.471) | 0.92 (0.916 - 0.924) |
|  |  |  | LogisticRegression | 112 | 0.074 | 0.886 (0.883 - 0.889) | 0.821 (0.818 - 0.824) | 0.316 (0.31 - 0.322) | 0.384 (0.38 - 0.389) | 0.463 (0.454 - 0.472) | 0.92 (0.916 - 0.924) |
|  |  |  | XGB_Imputed | 112 | 0.074 | 0.886 (0.883 - 0.889) | 0.839 (0.837 - 0.842) | 0.323 (0.318 - 0.328) | 0.402 (0.398 - 0.407) | 0.503 (0.494 - 0.513) | 0.917 (0.914 - 0.92) |
|  |  |  | **XGB_Unimputed** | 112 | 0.074 | 0.911 (0.909 - 0.913) | 0.853 (0.85 - 0.856) | 0.438 (0.431 - 0.444) | 0.461 (0.456 - 0.466) | 0.5 (0.49 - 0.51) | 0.944 (0.941 - 0.947) |
|  | **MORTALITY_10** | **1507** | LASSO | 182 | 0.121 | 0.825 (0.821 - 0.829) | 0.816 (0.814 - 0.818) | 0.39 (0.386 - 0.394) | 0.434 (0.431 - 0.437) | 0.546 (0.535 - 0.556) | 0.864 (0.857 - 0.87) |
|  |  |  | LogisticRegression | 182 | 0.121 | 0.824 (0.82 - 0.828) | 0.817 (0.815 - 0.819) | 0.391 (0.386 - 0.395) | 0.434 (0.431 - 0.438) | 0.548 (0.538 - 0.559) | 0.862 (0.856 - 0.868) |
|  |  |  | XGB_Imputed | 182 | 0.121 | 0.833 (0.83 - 0.836) | 0.824 (0.822 - 0.826) | 0.401 (0.397 - 0.406) | 0.463 (0.46 - 0.466) | 0.588 (0.58 - 0.597) | 0.867 (0.863 - 0.871) |
|  |  |  | **XGB_Unimputed** | 182 | 0.121 | 0.859 (0.857 - 0.862) | 0.842 (0.84 - 0.844) | 0.481 (0.476 - 0.486) | 0.496 (0.493 - 0.5) | 0.566 (0.557 - 0.574) | 0.9 (0.896 - 0.904) |
| **MSH > OH** | **CRITICAL_3** | **2201** | LASSO | 414 | 0.188 | 0.756 | 0.746 | 0.45 | 0.461 | 0.556 | 0.802 |
|  |  |  | LogisticRegression | 414 | 0.188 | 0.751 | 0.752 | 0.442 | 0.448 | 0.536 | 0.801 |
|  |  |  | XGB_Imputed | 414 | 0.188 | 0.714 | 0.765 | 0.488 | 0.478 | 0.698 | 0.717 |
|  |  |  | **XGB_Unimputed** | 414 | 0.188 | 0.671 | 0.781 | 0.514 | 0.458 | 0.739 | 0.655 |
|  | **CRITICAL_5** | **2201** | LASSO | 569 | 0.259 | 0.724 | 0.763 | 0.536 | 0.549 | 0.650 | 0.749 |
|  |  |  | LogisticRegression | 569 | 0.259 | 0.7 | 0.743 | 0.502 | 0.525 | 0.643 | 0.719 |
|  |  |  | XGB_Imputed | 569 | 0.259 | 0.632 | 0.78 | 0.567 | 0.536 | 0.821 | 0.566 |
|  |  |  | **XGB_Unimputed** | 569 | 0.259 | 0.743 | 0.789 | 0.565 | 0.568 | 0.654 | 0.775 |
|  | **CRITICAL_7** | **2201** | LASSO | 682 | 0.31 | 0.711 | 0.79 | 0.619 | 0.608 | 0.723 | 0.705 |
|  |  |  | LogisticRegression | 682 | 0.31 | 0.73 | 0.772 | 0.594 | 0.559 | 0.553 | 0.809 |
|  |  |  | **XGB_Imputed** | 682 | 0.31 | 0.742 | 0.807 | 0.655 | 0.645 | 0.758 | 0.735 |
|  |  |  | XGB_Unimputed | 682 | 0.31 | 0.74 | 0.801 | 0.642 | 0.622 | 0.691 | 0.762 |
|  | **CRITICAL_10** | **2201** | LASSO | 777 | 0.353 | 0.689 | 0.81 | 0.702 | 0.65 | 0.816 | 0.620 |
|  |  |  | LogisticRegression | 777 | 0.353 | 0.691 | 0.796 | 0.669 | 0.652 | 0.822 | 0.619 |
|  |  |  | **XGB_Imputed** | 777 | 0.353 | 0.746 | 0.816 | 0.711 | 0.672 | 0.739 | 0.749 |
|  |  |  | XGB_Unimputed | 777 | 0.353 | 0.74 | 0.808 | 0.69 | 0.649 | 0.680 | 0.773 |
|  | **MORTALITY_3** | **2201** | LASSO | 135 | 0.061 | 0.891 | 0.791 | 0.242 | 0.298 | 0.378 | 0.891 |
|  |  |  | LogisticRegression | 135 | 0.061 | 0.907 | 0.778 | 0.224 | 0.311 | 0.341 | 0.907 |
|  |  |  | XGB_Imputed | 135 | 0.061 | 0.929 | 0.722 | 0.17 | 0.103 | 0.067 | 0.929 |
|  |  |  | **XGB_Unimputed** | 135 | 0.061 | 0.936 | 0.877 | 0.444 | 0.417 | 0.370 | 0.936 |
|  | **MORTALITY_5** | **2201** | LASSO | 276 | 0.125 | 0.849 | 0.829 | 0.412 | 0.462 | 0.518 | 0.849 |
|  |  |  | LogisticRegression | 276 | 0.125 | 0.871 | 0.816 | 0.397 | 0.389 | 0.326 | 0.871 |
|  |  |  | XGB_Imputed | 276 | 0.125 | 0.761 | 0.805 | 0.366 | 0.419 | 0.688 | 0.761 |
|  |  |  | **XGB_Unimputed** | 276 | 0.125 | 0.873 | 0.862 | 0.523 | 0.513 | 0.533 | 0.873 |
|  | **MORTALITY_7** | **2201** | LASSO | 382 | 0.174 | 0.818 | 0.828 | 0.498 | 0.462 | 0.450 | 0.818 |
|  |  |  | LogisticRegression | 382 | 0.174 | 0.831 | 0.82 | 0.489 | 0.48 | 0.448 | 0.831 |
|  |  |  | XGB_Imputed | 382 | 0.174 | 0.174 | 0.83 | 0.494 | 0.296 | 1.000 | 0.174 |
|  |  |  | **XGB_Unimputed** | 382 | 0.174 | 0.841 | 0.856 | 0.583 | 0.578 | 0.628 | 0.841 |
|  | **MORTALITY_10** | **2201** | LASSO | 494 | 0.224 | 0.79 | 0.833 | 0.576 | 0.542 | 0.555 | 0.858 |
|  |  |  | LogisticRegression | 494 | 0.224 | 0.774 | 0.822 | 0.559 | 0.561 | 0.644 | 0.812 |
|  |  |  | XGB_Imputed | 494 | 0.224 | 0.772 | 0.833 | 0.598 | 0.579 | 0.698 | 0.793 |
|  |  |  | **XGB_Unimputed** | 494 | 0.224 | 0.787 | 0.844 | 0.638 | 0.589 | 0.680 | 0.818 |
| **MSH > PROSPECTIVE MSH** | **CRITICAL_3** | **175** | LASSO | 25 | 0.143 | 0.84 | 0.753 | 0.418 | 0.391 | 0.360 | 0.84 |
|  |  |  | LogisticRegression | 25 | 0.143 | 0.846 | 0.725 | 0.403 | 0.471 | 0.480 | 0.846 |
|  |  |  | XGB_Imputed | 25 | 0.143 | 0.869 | 0.771 | 0.497 | 0.511 | 0.480 | 0.869 |
|  |  |  | **XGB_Unimputed** | 25 | 0.143 | 0.76 | 0.781 | 0.533 | 0.4 | 0.560 | 0.76 |
|  | **CRITICAL_5** | **165** | LASSO | 29 | 0.176 | 0.806 | 0.685 | 0.375 | 0.407 | 0.379 | 0.806 |
|  |  |  | LogisticRegression | 29 | 0.176 | 0.764 | 0.655 | 0.369 | 0.316 | 0.310 | 0.764 |
|  |  |  | XGB_Imputed | 29 | 0.176 | 0.727 | 0.706 | 0.472 | 0.416 | 0.552 | 0.727 |
|  |  |  | **XGB_Unimputed** | 29 | 0.176 | 0.812 | 0.747 | 0.539 | 0.456 | 0.448 | 0.812 |
|  | **CRITICAL_7** | **158** | LASSO | 28 | 0.177 | 0.778 | 0.696 | 0.379 | 0.407 | 0.429 | 0.778 |
|  |  |  | LogisticRegression | 28 | 0.177 | 0.81 | 0.674 | 0.39 | 0.375 | 0.321 | 0.81 |
|  |  |  | **XGB_Imputed** | 28 | 0.177 | 0.778 | 0.726 | 0.419 | 0.407 | 0.429 | 0.778 |
|  |  |  | XGB_Unimputed | 28 | 0.177 | 0.772 | 0.717 | 0.401 | 0.438 | 0.500 | 0.772 |
|  | **CRITICAL_10** | **149** | LASSO | 28 | 0.188 | 0.785 | 0.722 | 0.48 | 0.484 | 0.536 | 0.785 |
|  |  |  | LogisticRegression | 28 | 0.188 | 0.718 | 0.71 | 0.412 | 0.4 | 0.500 | 0.718 |
|  |  |  | XGB_Imputed | 28 | 0.188 | 0.785 | 0.741 | 0.441 | 0.429 | 0.429 | 0.785 |
|  |  |  | **XGB_Unimputed** | 28 | 0.188 | 0.745 | 0.766 | 0.453 | 0.387 | 0.429 | 0.745 |
|  | **MORTALITY_3** | **175** | LASSO | 2 | 0.011 | 0.949 | 0.442 | 0.01 | 0 | 0.000 | 0.949 |
|  |  |  | LogisticRegression | 2 | 0.011 | 0.954 | 0.488 | 0.011 | 0 | 0.000 | 0.954 |
|  |  |  | XGB_Imputed | 2 | 0.011 | 0.989 | 0.827 | 0.036 | 0 | 0.000 | 0.989 |
|  |  |  | **XGB_Unimputed** | 2 | 0.011 | 0.971 | 0.962 | 0.551 | 0.286 | 0.500 | 0.971 |
|  | **MORTALITY_5** | **165** | LASSO | 4 | 0.024 | 0.903 | 0.713 | 0.043 | 0 | 0.000 | 0.903 |
|  |  |  | LogisticRegression | 4 | 0.024 | 0.945 | 0.668 | 0.035 | 0 | 0.000 | 0.945 |
|  |  |  | XGB_Imputed | 4 | 0.024 | 0.861 | 0.818 | 0.086 | 0.148 | 0.500 | 0.861 |
|  |  |  | **XGB_Unimputed** | 4 | 0.024 | 0.915 | 0.847 | 0.315 | 0.125 | 0.250 | 0.915 |
|  | **MORTALITY_7** | **158** | LASSO | 6 | 0.038 | 0.911 | 0.825 | 0.149 | 0.3 | 0.500 | 0.911 |
|  |  |  | LogisticRegression | 6 | 0.038 | 0.918 | 0.811 | 0.162 | 0.235 | 0.333 | 0.918 |
|  |  |  | XGB_Imputed | 6 | 0.038 | 0.038 | 0.845 | 0.185 | 0.073 | 1.000 | 0.038 |
|  |  |  | **XGB_Unimputed** | 6 | 0.038 | 0.911 | 0.9 | 0.354 | 0.3 | 0.500 | 0.911 |
|  | **MORTALITY_10** | **149** | LASSO | 8 | 0.054 | 0.899 | 0.853 | 0.41 | 0.348 | 0.500 | 0.899 |
|  |  |  | LogisticRegression | 8 | 0.054 | 0.859 | 0.822 | 0.316 | 0.276 | 0.500 | 0.859 |
|  |  |  | XGB_Imputed | 8 | 0.054 | 0.872 | 0.884 | 0.499 | 0.345 | 0.625 | 0.872 |
|  |  |  | **XGB_Unimputed** | 8 | 0.054 | 0.893 | 0.925 | 0.468 | 0.429 | 0.750 | 0.893 |
| **MSH > PROSPECTIVE OH** | **CRITICAL_3** | **208** | LASSO | 34 | 0.163 | 0.808 | 0.655 | 0.334 | 0.375 | 0.353 | 0.808 |
|  |  |  | LogisticRegression | 34 | 0.163 | 0.779 | 0.657 | 0.354 | 0.303 | 0.294 | 0.779 |
|  |  |  | XGB_Imputed | 34 | 0.163 | 0.76 | 0.713 | 0.312 | 0.419 | 0.529 | 0.76 |
|  |  |  | **XGB_Unimputed** | 34 | 0.163 | 0.755 | 0.739 | 0.357 | 0.463 | 0.647 | 0.755 |
|  | **CRITICAL_5** | **201** | LASSO | 34 | 0.169 | 0.761 | 0.67 | 0.328 | 0.385 | 0.441 | 0.761 |
|  |  |  | LogisticRegression | 34 | 0.169 | 0.726 | 0.648 | 0.312 | 0.321 | 0.382 | 0.726 |
|  |  |  | XGB_Imputed | 34 | 0.169 | 0.692 | 0.713 | 0.329 | 0.415 | 0.647 | 0.692 |
|  |  |  | **XGB_Unimputed** | 34 | 0.169 | 0.781 | 0.757 | 0.374 | 0.436 | 0.500 | 0.781 |
|  | **CRITICAL_7** | **196** | LASSO | 38 | 0.194 | 0.73 | 0.689 | 0.363 | 0.391 | 0.447 | 0.73 |
|  |  |  | LogisticRegression | 38 | 0.194 | 0.76 | 0.661 | 0.358 | 0.356 | 0.342 | 0.76 |
|  |  |  | XGB_Imputed | 38 | 0.194 | 0.74 | 0.751 | 0.45 | 0.452 | 0.553 | 0.74 |
|  |  |  | **XGB_Unimputed** | 38 | 0.194 | 0.73 | 0.763 | 0.458 | 0.485 | 0.658 | 0.73 |
|  | **CRITICAL_10** | **187** | LASSO | 41 | 0.219 | 0.722 | 0.741 | 0.461 | 0.469 | 0.561 | 0.722 |
|  |  |  | LogisticRegression | 41 | 0.219 | 0.706 | 0.706 | 0.453 | 0.455 | 0.561 | 0.706 |
|  |  |  | XGB_Imputed | 41 | 0.219 | 0.733 | 0.766 | 0.485 | 0.444 | 0.488 | 0.733 |
|  |  |  | **XGB_Unimputed** | 41 | 0.219 | 0.738 | 0.767 | 0.499 | 0.505 | 0.610 | 0.738 |
|  | **MORTALITY_3** | **208** | LASSO | 3 | 0.014 | 0.947 | 0.539 | 0.259 | 0.154 | 0.333 | 0.947 |
|  |  |  | LogisticRegression | 3 | 0.014 | 0.962 | 0.506 | 0.342 | 0.2 | 0.333 | 0.962 |
|  |  |  | XGB_Imputed | 3 | 0.014 | 0.981 | 0.664 | 0.068 | 0 | 0.000 | 0.981 |
|  |  |  | **XGB_Unimputed** | 3 | 0.014 | 0.942 | 0.879 | 0.131 | 0.143 | 0.333 | 0.942 |
|  | **MORTALITY_5** | **201** | LASSO | 5 | 0.025 | 0.93 | 0.818 | 0.225 | 0.222 | 0.400 | 0.93 |
|  |  |  | LogisticRegression | 5 | 0.025 | 0.965 | 0.739 | 0.127 | 0.222 | 0.200 | 0.965 |
|  |  |  | XGB_Imputed | 5 | 0.025 | 0.806 | 0.807 | 0.06 | 0.133 | 0.600 | 0.806 |
|  |  |  | **XGB_Unimputed** | 5 | 0.025 | 0.93 | 0.825 | 0.307 | 0.222 | 0.400 | 0.93 |
|  | **MORTALITY_7** | **196** | LASSO | 11 | 0.056 | 0.898 | 0.676 | 0.255 | 0.231 | 0.273 | 0.898 |
|  |  |  | LogisticRegression | 11 | 0.056 | 0.903 | 0.613 | 0.201 | 0.174 | 0.182 | 0.903 |
|  |  |  | **XGB_Imputed** | 11 | 0.056 | 0.056 | 0.7 | 0.21 | 0.106 | 1.000 | 0.056 |
|  |  |  | XGB_Unimputed | 11 | 0.056 | 0.862 | 0.685 | 0.303 | 0.27 | 0.455 | 0.862 |
|  | **MORTALITY_10** | **187** | **LASSO** | 15 | 0.08 | 0.856 | 0.731 | 0.319 | 0.341 | 0.467 | 0.856 |
|  |  |  | LogisticRegression | 15 | 0.08 | 0.824 | 0.719 | 0.272 | 0.327 | 0.533 | 0.824 |
|  |  |  | XGB_Imputed | 15 | 0.08 | 0.759 | 0.66 | 0.203 | 0.262 | 0.533 | 0.759 |
|  |  |  | XGB_Unimputed | 15 | 0.08 | 0.765 | 0.717 | 0.301 | 0.267 | 0.533 | 0.765 |
