# Supplementary material for: Machine Learning to Predict Mortality and Critical Events in a Cohort of Patients With COVID-19 in New York City: Model Development and Validation
Source: J Med Internet Res. 2020 Nov 6;22(11):e24018. doi: 10.2196/24018 (PMC7652593; doi:10.2196/24018)
Supplement: Multimedia Appendix 7 [file jmir_v22i11e24018_app7.docx]

**Supplementary Table 6:** Clinical features ranked in decreasing order based on their significance (most significant predictor= 1, least significant predictor=10) for Critical Event and Mortality at 7 Days

| **Outcome (Critical Event or Mortality)** | **Clinical Features** | **Rank** |
| --- | --- | --- |
| Critical Event at 7 Days | Acute Kidney Injury (AKI) | 1 |
|  | Lactate Dehydrogenase (LDH) | 2 |
|  | Respirations | 3 |
|  | Glucose | 4 |
|  | Diastolic Blood Pressure | 5 |
|  | C-Reactive Protein | 6 |
|  | pH | 7 |
|  | Total Protein | 8 |
|  | D-Dimer | 9 |
|  | Systolic Blood Pressure | 10 |
|  | | |
| Mortality at 7 Days | Age | 1 |
|  | Anion Gap | 2 |
|  | C-Reactive Protein | 3 |
|  | Lactate Dehydrogenase (LDH) | 4 |
|  | Oxygen Saturation | 5 |
|  | Blood Urea Nitrogen | 6 |
|  | Ferritin | 7 |
|  | Red Cell Distribution Width (RDW) | 8 |
|  | Diastolic Blood Pressure | 9 |
|  | Lactate | 10 |
